# Polarity effects in 4-fluoro- and 4-(trifluoromethyl)prolines

**DOI:** 10.3762/bjoc.16.151

**Published:** 2020-07-23

**Authors:** Vladimir Kubyshkin

**Affiliations:** 1 University of Manitoba, Dysart Rd. 144, Winnipeg, R3T 2N2, Canada

**Keywords:** amino acids, *cis*–*trans* isomerism, fluorine, polarity, proline

## Abstract

Fluorine-containing analogues of proline are valuable tools in engineering and NMR spectroscopic studies of peptides and proteins. Their use relies on the fundamental understanding of the interplay between the substituents and the main chain groups of the amino acid residue. This study aims to showcase the polarity-related effects that arise from the interaction between the functional groups in molecular models. Properties such as conformation, acid–base transition, and amide-bond isomerism were examined for diastereomeric 4-fluoroprolines, 4-(trifluoromethyl)prolines, and 1,1-difluoro-5-azaspiro[2.4]heptane-6-carboxylates. The preferred conformation on the proline ring originated from a preferential axial positioning for a single fluorine atom, and an equatorial positioning for a trifluoromethyl- or a difluoromethylene group. This orientation of the substituents explains the observed trends in the p*K*_a_ values, lipophilicity, and the kinetics of the amide bond rotation. The study also provides a set of evidences that the transition state of the amide-bond rotation in peptidyl-prolyl favors C^4^-*exo* conformation of the pyrrolidine ring.

## Introduction

Polarity is among the key features essential for understanding the behavior of organic molecules of biological origin. In particular, there is a set of polarity-related issues in the chemistry of amino acids, while the latter are key actors in multiple biochemical processes. In protein translation, for example, 20 (+2) amino acids are utilized for polymerizing them into a primary structure of a protein. Most of these structures share the same key elements with the structure of alanine, which constitute the backbone features. The classification of the amino acid residues by hydrophobic/hydrophilic usually refers to the variable part, which locates in the side chain. The introduction of an aliphatic or an aromatic group into the side chain usually renders an amino acid hydrophobic, while the introduction of a polar or an ionizable group makes it hydrophilic.

**Figure 1:**
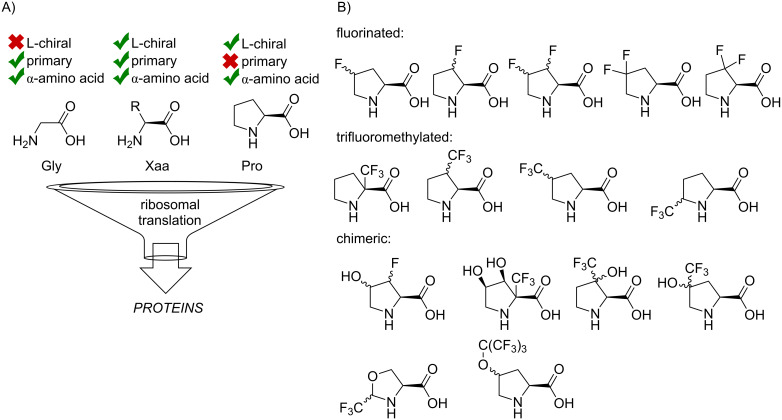
A) Three types of the backbone amino acid structures that are included in protein translation: glycine, alanine, and the set of its structural derivatives, proline. B) The portfolio of the fluorinated amino acids developed to date.

Two amino acids stand out from the dualistic hydrophilic/hydrophobic classification: glycine and proline (Figure 1A) [1]. The origin of their effect onto the structure polarity is not due to the presence of additional functional groups, but due to the altered backbone folding and solvation. For example, a proline residue cannot be considered hydrophobic, even though, it contains the same number of carbon atoms as valine, which is evidently hydrophobic [2]. In fact, a proline contribution to the peptide polarity can be ambivalent and mainly depends on the underlying secondary structure. The lack of a polar N–H bond in peptidyl-prolyl can decrease the polarity in one case, and increase in another. The first scenario may occur if a free N–H bond would otherwise be exposed to the solvent. Proline lacks an N–H bond, thus the polarity drops down. The second scenario would occur if there was an N–H^…^O=C hydrogen bond in the structure. Thus, the presence of proline would leave an unsolvated carbonyl group, and its appearance leads to a polarity rise. Finally, a proline residue usually prefers extended secondary fold and may identify structural breaks in a sequence. This fact contributes to the reputation of proline as a polar residue [3–5].

There is a rich portfolio of fluorine-containing proline analogues that have been developed to date (Figure 1B): fluorinated [6–12], trifluoromethylated [13–23], chimeric [16,19,24–29], conformationally restricted [30–33] having variations in the ring size [34–39], non-α [40–44], and other analogues [45–48]. The fluorine-containing functional groups are usually chemically inert under most biologically relevant conditions. The presence of these groups adjacent to the proline structure helps to modulate the conformational landscape of the parent amino acid, and this effectively alters the folding of the peptide chain when an analogue is included in it as a residue [49–50]. The presence of a fluorine-rich group in the structure is also beneficial for the NMR studies based on the detection of the ^19^F nucleus [51–53]. Limited attention has been given to the polarity effects in the proline analogues though. Few studies reported peptides containing proline analogues with distinct hydrophobic properties can impact their ability to pass biomembranes [54–56]. A crystallographic study has shown that when included into a protein structure, a fluorine atom exhibits a network of interaction within a protein core [57]. Another recent study showed that the substitution of proline with analogues can result in the conformational stabilization and polarity effects competing with each other [58]. Finally, it has been shown that fluoroprolines can alter donor–acceptor interactions of the proline ring with a tryptophan residue [59].

**Figure 2:**
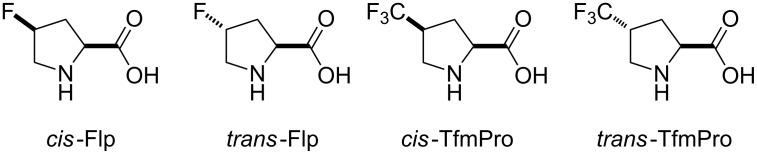
The set of amino acids examined in this study.

All these findings indicate that polarity effects should be taken into account in the characterization of proline analogues containing fluorinated groups. Nonetheless, most studies focus on conformational properties of the analogues, and much less is known about their polarity. This study aims to shed light on the polarity-related phenomena in two typical examples: 4-fluoroprolines and 4-(trifluoromethyl)prolines (Figure 2). The study aims to provide experimental characterization of these amino acids in the context of simple molecular models. Thereby, it may help to build predictions for the amino acid use in the context of more complex structures: peptides and proteins.

## Results and Discussion

### Model compounds

**Figure 3:**
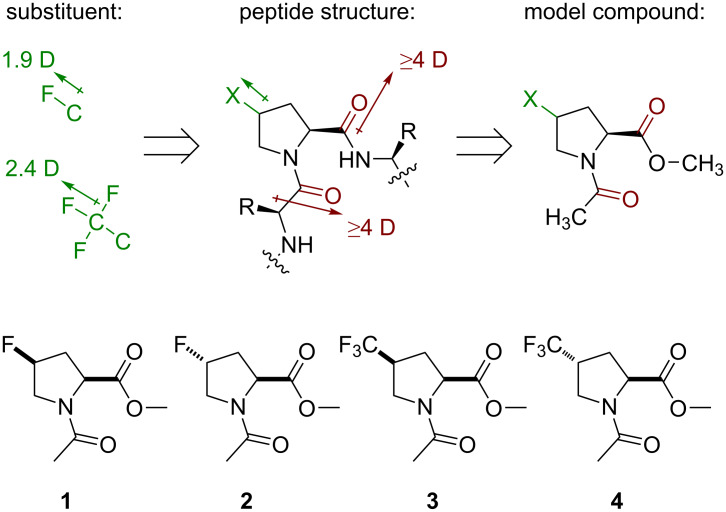
Design of the model system.

The study was originally set up following an assumption that a peptide containing a proline analogue would form a system of three dipoles. The peptide bond itself creates a strong dipole, with a direction that roughly aligns the direction of the carbonyl bond (see Figure S1 in Supporting Information File 1 for the estimation of dipole size and orientation). A proline residue is surrounded by two such dipoles originated from the up- and downstream peptide bonds. The strength of the dipoles can be estimated as 4–5 D. A fluorine-containing substituent adds a new dipole to the system. A C–F dipole is estimated as 1.9 D, whereas a CF_3_-group dipole is about 2.4 D [60]. Thus, a proline analogue that contains these substituents would form a system of three mutually interacting dipoles (Figure 3).

To mimic this situation, a conventional model was chosen: the methyl ester of an *N*-acetylated amino acid. This model is very convenient, because it maintains the set of the dipoles. At the same time, it lacks the N–H bonds, which would otherwise disturb the measurements in nonpolar solvents due to the formation of specific hydrogen-bonded structures, such as γ- and δ-turns (also called C7- and C5-bonds, respectively) [61–62]. Thus, it was decided to examine the model compounds **1**–**4** and molecular effects therein.

### Conformation of the proline ring

**Figure 4:**
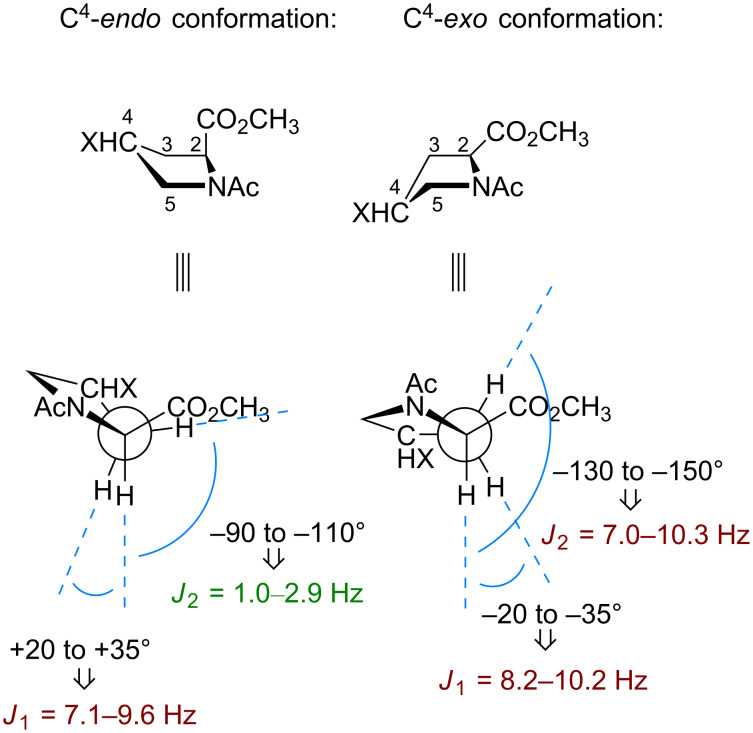
Propagation of the C^4^-conformation into the values of the *J* coupling in the C^2^H–C^3^H_2_ fragments.

Due to its cyclic nature, the pyrrolidine ring in proline can adopt a few distinct conformational states [61,63]. The envelope conformation of the 5-membered pyrrolidine ring is commonly assumed to transit between two situations: 1) the one in which the C^4^-atom is displaced from the main plain in the same direction as the carboxylic group (C^4^-*endo* or *down* pucker), and 2) the one with the displacement in the opposite direction (C^4^-*exo* or *up* pucker). Unsubstituted proline does not have a distinct preference towards either form, and the transition between them occurs in a relatively fast kinetic mode (barrier 10–13 kJ mol^−1^) [64–65]. The 2-CH multiplicity in the ^1^H NMR spectrum is indicative of the conformation adopted by the ring. A C^4^-*endo* conformation exhibits two vicinal *J* couplings with one value being large and one being small, whereas a C^4^-*exo* conformation displays two large *J* couplings [66] (Figure 4).

A substituent at the C^4^-atom creates a certain preference towards one or another conformation. An electron-withdrawing substituent tends to adopt an axial position due to an orbital effect called the *gauche*-effect (stereoelectronic effect) [67–68]. Conversely, a bulky substituent adopts an equatorial position (steric effect) [69–70]. At the same time, there has been no evidence that a direct dipole–dipole interaction between the substituent and the main chain groups could have an effect onto the conformation.

**Figure 5:**
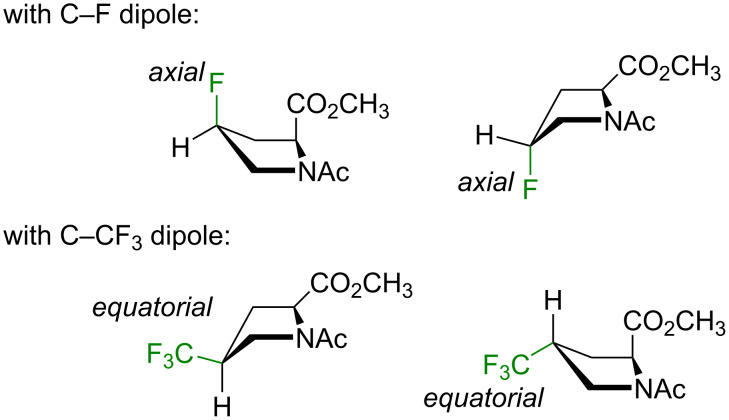
Preferred side-chain conformations according to the multiplicity data.

Here, the NMR data for the model compounds **1**–**4** were collected in three solvents with distinct dielectric properties (Table 1). The results were interpreted by comparing the experimental 2-CH multiplicity with the one predicted for the pure conformers (Figure 4). As can be seen from the data, both fluoroprolines exhibited stabilization of certain side-chain conformers: the *cis*-isomer **1** stabilized the C^4^-*endo* envelope, whereas the *trans*-isomer **2** stabilized the C^4^-*exo* (Figure 5). The major ring conformation (C^4^-*endo* for **1** and C^4^-*exo* for **2**) persisted in all three examined solvents and in both amide rotameric states. This outcome is fully consistent with the literature data [68], and it agrees with two conclusions: 1) the fluorine atom adopts an axial conformation and 2) the origin of the effect is orbital (the *gauche*-effect) rather than through space dipolar interaction (assuming that an interaction of dipoles would be attenuated in polar solvents, which was not observed).

**Table 1:** 2-CH multiplicity data and the conclusions regarding the C^4^ conformation in the model compounds.^a^

compound	^1^H NMR multiplicity of 2-CH^b^								
	in CDCl_3_, ε 4.8			in CD_2_Cl_2_, ε 8.9			in D_2_O, ε 80.1		
	*J*_1_, Hz	*J*_2_, Hz	C^4^-	*J*_1_, Hz	*J*_2_, Hz	C^4^-	*J*_1_, Hz	*J*_2_, Hz	C^4^-

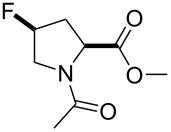 **1**	9.8 (9.4)	1.0 (n.d.)^c^	*endo* *(endo)*	9.8 (9.5)	1.0 (n.d.)^c^	*endo* *(endo)*	8.8 (9.6)	2.9 (n.d.)^c^	*endo* *(endo)*
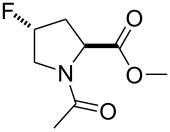 **2**	8.5 (8.4)	8.5 (8.4)	*exo* *(exo)*	8.5 (8.0)	8.5 (8.0)	*exo* *(exo)*	10.0 (8.4)	7.8 (8.4)	*exo* *(exo)*
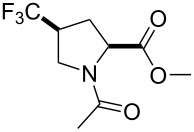 **3**	8.3 (9.0)	8.3 (5.4)	*exo* *(mix)*^d^	8.3 (9.2)	8.3 (5.2)	*exo* *(mix)*^d^	8.3 (9.9)	8.3 (4.1)	*exo* *(mix)*^d^
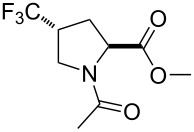 **4**	9.0 (8.7)	2.3 (1.7)	*endo* *(endo)*	9.0 (8.5)	2.5 (1.6)	*endo* *(endo)*	9.2 (8.6)	4.0 (2.6)	*mix*^d^ *(endo)*

^a^Read out from 1D ^1^H NMR spectra recorded at 700 or 500 MHz frequency at 298 K; ^b^the s-*trans* (major) amide rotamer data is shown first; the results in parentheses are for the s-*cis* (minor) amide rotamer; ^c^n.d. not detected, the resonance appears as a doublet; ^d^mix = a mixture of two conformations.

A stabilization of certain side-chain conformers was also observed in the trifluoromethylated analogues, **3** and **4**. However, the effect was not entirely clean. For example, in the *cis*-isomer **3**, the *exo*-C^4^ envelope was only preferred in the major amide rotamer, whereas in the minor one, a mixture of two envelopes was observed. In the *trans*-isomer **4**, the C^4^-*endo* envelope was found in all cases, except for the major amide rotamer in water, where the conformer was less defined. Overall, these outcomes demonstrate that the CF_3_ group favors an equatorial placement, but the preference is notably weaker as compared to the monofluoroprolines (Figure 5).

It could be speculated that if the dipolar interaction of the polar substituents with the main chain groups would impact the side-chain conformation, this effect would be sensitive to the polarity of the solvent. The fact that the solvent plays only a minor role demonstrates that stronger determinants exist in the structure, namely, the stereoelectronic (C–F) and steric (C–CF_3_) effects from the substituents.

### Acid–base transition

**Figure 6:**
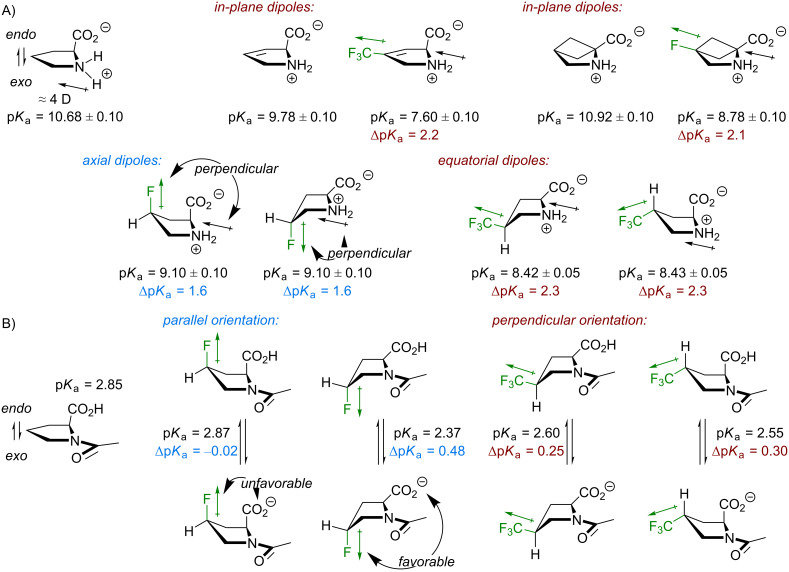
A) The basicity reduction from the introduction of the dipoles reflects the preferred conformation of the side chains. The positive end of the ammonium dipole is on the hydrogen atoms attached to the nitrogen. The nitrogen atom is the negative end. B) The acidity of the carboxylic group depends on the relative orientation of the substituent dipole with respect to the charged carboxylate. Only the s-*cis* rotamer data is shown. The error is ±0.05.

The orientation of the substituents can also be inferred from the data on basicity of the ammonium group in free amino acids. It has been already mentioned that both, C–F and CF_3_ groups introduce dipoles of similar sizes into the molecules. The latter one is slightly larger, but it is also one bond more distant to the main chain groups (Figure 3). Effectively, these dipoles reduce the basicity due to the interaction with the ammonium dipole through bond and through space [71] (Figure 6A). It can be seen from the experimental data that the basicity reduction from substituents that are parallel and perpendicular to the pyrrolidine ring are different. There are examples showing that the C–F or C–CF_3_ fragment in plane with the ammonium dipole reduces the basicity by about Δp*K*_a_ ≈ 2.2 [72–73]. The same value was obtained for (trifluoromethyl)prolines (Δp*K*_a_ ≈ 2.3) with equatorially placed C–CF_3_. In the fluoroprolines, there is a distinctly different orientation of the C–F dipoles, and the basicity reduction is notably smaller (Δp*K*_a_ ≈ 1.6). Here, the substituent dipole is nearly perpendicular to the ammonium one, and this weakens their interaction.

Similar considerations can be applied to the acidity of the carboxylic group (Figure 6B), where the mutual orientation of the C–X dipole and the carboxylate -CO_2_^−^ charge has an effect [74]. In diastereomeric fluoroprolines there is a large difference in the mutual orientation of the groups, while in the (trifluoromethyl)prolines the orientation is similar. This is reflected in the acidity values.

### Lipophilicity

Another parameter, which may be sensitive to the orientation of the dipoles within a molecule is the lipophilicity. It is well known that single aliphatic fluorination usually increases polarity of a molecule due the newly introduced polar C–F bond. A CF_3_ group introduces a dipole of a similar size (see Figure 2), however, due to its high molar volume it increases the hydrophobicity of a molecule [75]. The overall outcome may appear paradoxical: a CF_3_ group can make a molecule more polar, but also more lipophilic at the same time. In addition, interactions of these dipoles with the ones preexisting in the parent molecule should always be considered for predicting the molecular polarity [60,76].

Experimental lipophilicity values found for the model compounds **1**–**4** fully corroborate the expectations: the monofluorinated compounds **1** and **2** exhibited a slightly lower log*P* compared to unsubstituted reference (Δlog*P*_H/F_ ≈ −0.2 to −0.4), whereas in the trifluoromethylated compounds **3** and **4** the log*P* was notably higher (Δlog*P*_H/CF3_ ≈ +0.7). Interestingly, the diastereomers exhibited some differences. The *cis*-diastereomer **1** with the C–F bond pointing in the same direction as the carboxymethyl group, appeared more polar compared to the *trans*-isomer **2**, where the direction was opposite (Δlog*P_cis/trans_* ≈ −0.18). The same effect was observed in 4-hydroxyprolines as well [13] (Δlog*P_cis/trans_* ≈ −0.19).

**Figure 7:**
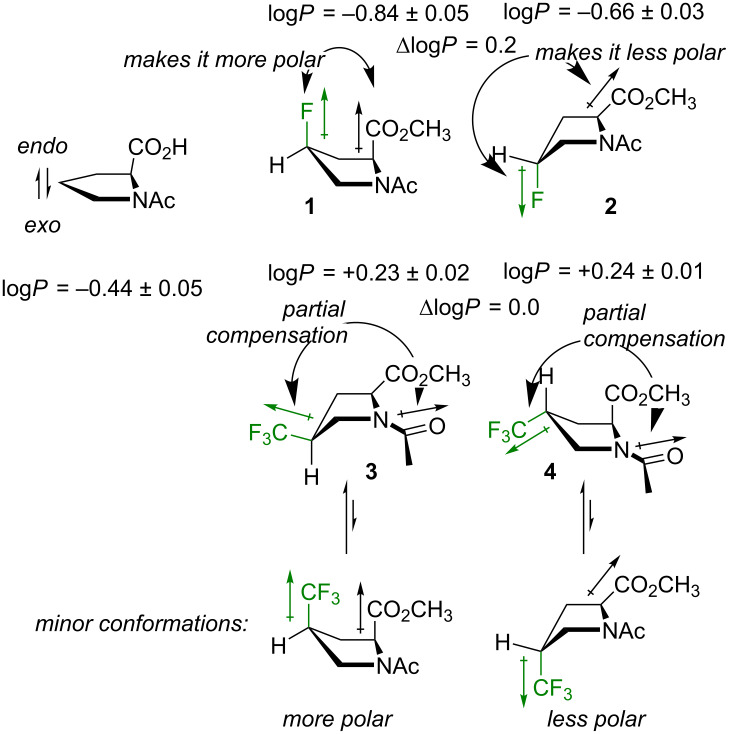
The lipophilicity data of the model compounds.

In contrast to **1** and **2**, the trifluoromethylated compounds **3** and **4** showed no difference between the lipophilicity of the diastereomers. This is due to the fact that the C–CF_3_ substituent orientation is equatorial, thus nearly perpendicular to the CH–CO_2_CH_3_, and the dipoles do not sufficiently interact in this situation. However, minor conformations with axial CF_3_ substituents do exist in the model compounds **3** and **4** (Figure 7). For example, both species were quite well separable using standard silica gel chromatography settings, where **4** appeared less polar than **3**. The existence of the minor conformations will also be relevant in the explanation of the amide-rotation barriers (vide infra).

It should be noted that calculation of the net dipole is of little aid when accessing polarity of the model compounds **1**–**4** [77]. For example, the dipole moment calculated for compound **1** (μ**_1_** = 4.1–4.9 D) appeared smaller compared to the one of compound **2** (μ**_2_** = 5.4–5.9 D), although an entirely opposite outcome would be expected looking at the lipophilicity data. At the same time, the relatively low net dipole for the compounds **3** and **4** (μ**_3,4_** = 1.4–2.1 D) predicts that there is an additional polarity drop associated with partial mutual compensation of the CF_3_ and the amide dipoles (Figure 7). Indeed, this occurs experimentally. In fact, the lipophilicity contribution from the CF_3_ group in **3** and **4** (Δlog*P*_H/CF3_ +0.7) is notably larger compared to an isolated CF_3_ group in a hydrocarbon chain (Δlog*P*_H/CF3_ +0.3 – +0.4) [78]. In spite of this outcome, the model system seems too complex to be described by a single polarity parameter such as net dipole. This is due to the fact that the system maintains some degree of conformational flexibility (e.g., rotation of the carboxymethyl group), which creates a conformational landscape rather than a single conformation. At the same time, a net dipole calculation fully neglects the distance between individual interacting dipoles, which is a very important parameter by itself (e.g., the energy of the dipole–dipole interaction is reverse proportional to the cubic distance).

Discussions based on the net (molecular) dipole have become quite popular in the recent literature. Indeed, this parameter is critically relevant for the understanding of relatively small and simple molecular fragments [60,75–76,78], e.g., an axially rotating CF_3_ group. Nonetheless, it is barely conclusive to approach interaction of complex molecules with solvents using net dipole alone. For example, even for the relatively simple molecules like **1**–**4**, the net dipole would not adequately represent neither the complexity of the interaction network within the molecule, nor probing of the molecular sites with relatively small dipoles of the solvent. This is why here it seems more reasonable to adhere to simple schematic descriptions such as the one shown in Figure 7.

### Amide-bond rotation: thermodynamics

Isomerization of the amide (peptide) bond is an important issue in protein chemistry. The peptide bond can exist in two discrete states, commonly designated as *trans*- and *cis*-rotamers (ω = 180 and 0°, respectively). The *cis*-peptide bond is very rare in natural proteins [79], except for the cases when it precedes a proline residue. The secondary amino group of proline forms a tertiary amide bond, which can often be found in either rotameric state. The peptide-bond isomerism in the peptidyl-prolyl fragments is involved in numerous biological processes in natural proteins [80].

**Figure 8:**
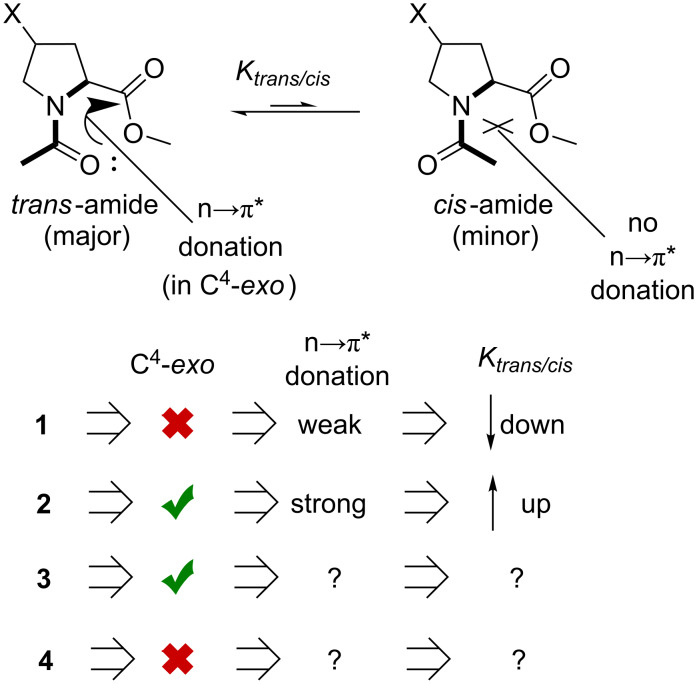
The expectations regarding the amide-bond rotation preferences in **1**–**4**.

Model compounds have been utilized to characterize the intrinsic propensity of proline and its analogues towards *trans*- and *cis*-amides [81]. An unsubstituted proline residue usually exhibits a preference to form a *trans*-amide. Fluoroprolines are notorious for the relative stabilization of the amide rotamers depending on the stereochemistry at the C^4^-atom. This is known as the chiral bias. The effect occurs indirectly due to the impact onto the side-chain envelope conformation: the C^4^-*exo* conformation creates a favorable relative arrangement of the main-chain groups. This energetic favorability is typically attributed to the n→π* donative interaction between the carbonyl groups, although the exact nature of this interaction (orbital or dipolar) is debatable [82]. Result of this effect though, is that the *trans*-fluoroproline shifts the equilibrium towards a higher abundance of the *trans*-amide, while *cis*-fluoroproline promotes the *cis*-amide (Figure 8). This was indeed found in the experimental data for the compounds **1** and **2** [83].

The expectation was that the trifluoromethylated amino acids would exhibit similar effects with an opposite chiral bias. However, experimentally it was found that the *trans/cis* ratio in both compounds **3** and **4** is quite similar to the one in the unsubstituted reference (Table 2). The value *K_trans/cis_* decreased in a row: unsubstituted > **3** > **4**. Nonetheless, these differences were rather small and did not exceed 1.1 kJ mol^−1^. Thus, (trifluoromethyl)prolines represent a peculiar case, where the C^4^-conformational stabilization is so weak, that it only has a minor influence onto the *trans/cis* ratio. In contrast, the *gauche*-effect in fluoroprolines causes a much stronger stabilization of the C^4^-conformations, as this propagates into stronger effects in the *trans/cis* equilibrium. In addition, there is a weak but systematic modulation in the *trans/cis* equilibrium caused by the solvent (*K*_benzene_ ≈ *K*_water_ > *K*_dichloromethane_). These are, however, still poorly understood and will not be further addressed here (see [74,77,84] for some discussions).

**Table 2:** Amide-rotation equilibrium in the model compounds.^a^

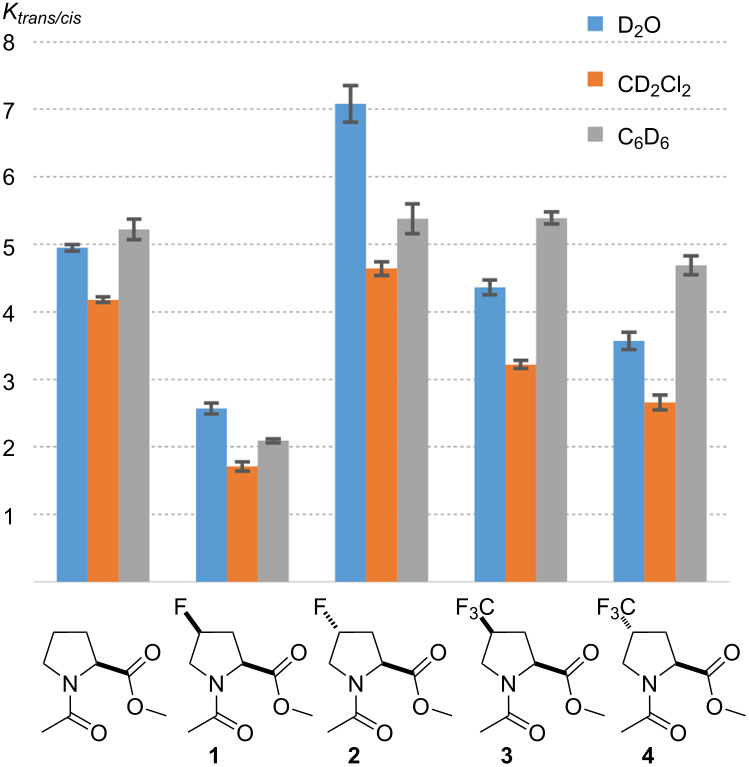			

compound	*K_trans/cis_*		
	in D_2_O, ε 80.1	in CD_2_Cl_2_, ε 8.9	in C_6_D_6_, ε 2.3

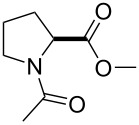	4.95 ± 0.05	4.18 ± 0.04	5.22 ± 0.15
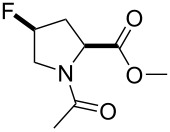 **1**	2.57 ± 0.08	1.71 ± 0.07	2.09 ± 0.03
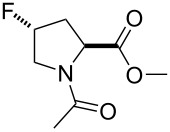 **2**	7.08 ± 0.27	4.64 ± 0.10	5.38 ± 0.22
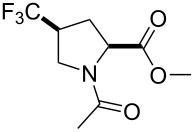 **3**	4.36 ± 0.11	3.22 ± 0.06	5.39 ± 0.09
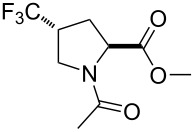 **4**	3.57 ± 0.13	2.66 ± 0.11	4.69 ± 0.14

^a^Measured by ^1^H and ^19^F NMR at 298 K. The values are provided with the standard deviation.

It is very well known that a proline-to-fluoroproline substitution alters protein folding. Predominantly, the effect onto the stability has been observed, and not the structure. The intrinsic preferences of the fluoroprolines translate into the differences in the folding energy and associated parameters such as melting points [81,85–90]. This study suggests that the analogous effect from (trifluoromethyl)prolines would be much weaker. The effects of their presence is a polypeptide structure should rather be associated with the increase in the molecular volume and hydrophobicity, and less with the backbone folding.

### Amide-bond rotation: kinetics

Amide-bond rotation is known as an intrinsically slow process, which contributes to the rate-limiting steps in the protein folding [91] and molecular timing phenomena [92]. In peptidyl-prolyl bonds, the transition between the rotational states *cis*-amide and *trans*-amide usually occurs in the mHz scale, with the barriers of rotation around 80–90 kJ mol^−1^ (in water). At the same time, the amide rotation is a simple process with only one (prevalent) transition state and two ground states [93]. The high level of the energetic barrier implies that there is only a negligible subpopulation of molecules that may be involved in the transition, and therefore the rotation should not necessarily proceed from the major conformation. This is an important precaution that should be kept in mind in the analysis of the data. In model compounds, the barrier of rotation can be determined relatively accurately with the experimental methods such as EXSY NMR [94]. However, the interpretation is not always straightforward. In fact, the barrier of rotation is perhaps the most complex parameter among all those presented in this work.

In the next step, the amide rotation was measured in compounds **1**–**4** in three solvents with different polarities (for a detailed procedure see Supporting Information File 1). To make the interpretation easier, only *cis*-to-*trans* barrier is presented (Table 3). The analysis shows that this parameter decreased in a row: unsubstituted > **1** > **2** > **4** > **3** in all examined solvents.

**Table 3:** Amide-rotation velocity in the model compounds.^a^

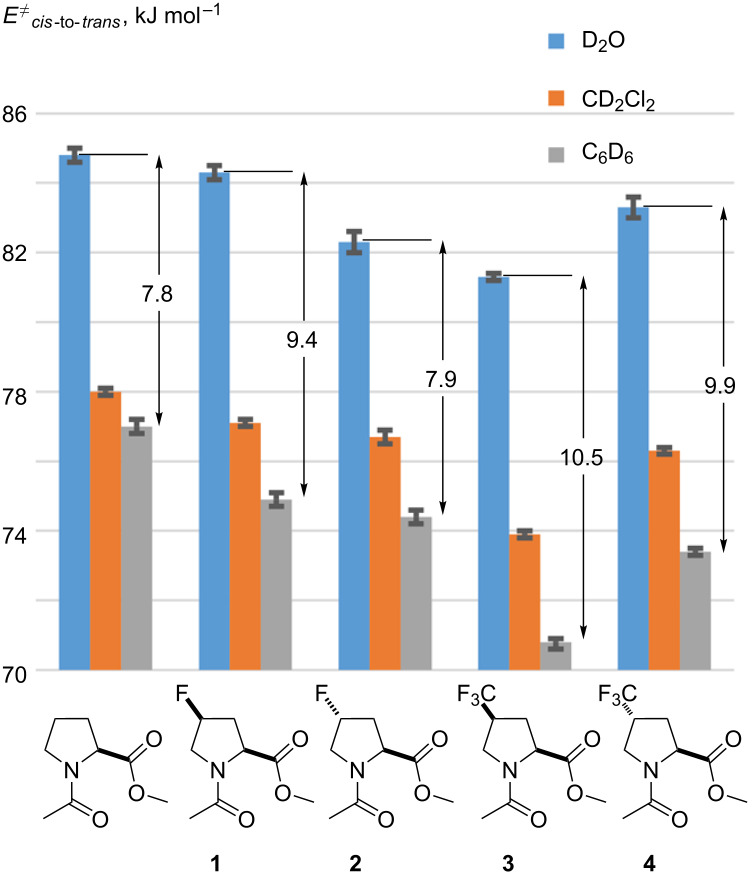			

compound	*k_cis_*_-to-_*_trans_*, Hz		
	in D_2_O, ε 80.1 (310 K)	in CD_2_Cl_2_, ε 8.9 (298 K)	in C_6_D_6_, ε 2.3 (298 K)

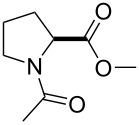	0.033 ± 0.002	0.033 ± 0.002^b^	0.043 ± 0.004
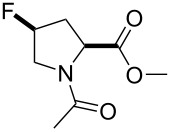 **1**	0.041 ± 0.004	0.187 ± 0.003	0.463 ± 0.034
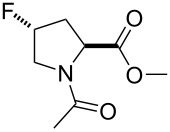 **2**	0.087 ± 0.009	0.220 ± 0.016	0.556 ± 0.027
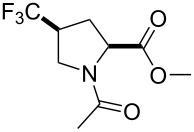 **3**	0.131 ± 0.008	0.690 ± 0.019	2.44 ± 0.07
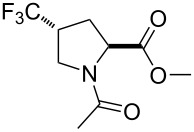 **4**	0.060 ± 0.006	0.265 ± 0.007	0.855 ± 0.031

^a^Measured by ^1^H and ^19^F EXSY NMR. Few spectra with different mixing times were measured. The exchange rate values were calculated from various exchange resonances and averaged. Their root mean deviation from the mean value is provided. The *k* values were converted to the energy barriers using the Eyring equation. The error in *E*^≠^ takes into account the standard deviation in the *k* values, and does not take into account the temperature calibration error. ^b^The value is for CDCl_3_, ε 4.8.

Three interesting observations should be analyzed based on the presented data. First, the order unsubstituted > fluoroproline > (trifluoromethyl)proline reflects the increasing electron-withdrawing effect of the substituents H > –F > –CF_3_, and it recapitulates the basicity trends observed previously (see Figure 6). Second, the solvation by polar solvents such as water substantially increases the stability of the ground-state amide bond, thereby elevating the rotation barrier [84,95]. Interestingly, the increase of the barrier when going from benzene to water (Δ_water/benzene_ = *E*^≠^_in water_ − E^≠^_in benzene_) was found larger in **1** compared to **2**, whereas in the diastereomeric couple **3** and **4** only a negligible difference was observed. This behavior can be explained by suggesting that the solvation of the diastereomers is also different. Because **1** is more polar than **2**, its desolvation in the course of the rotation process produces higher energetic costs. This can be seen from the fact that the energetic difference (1.5 kJ mol^−1^) is similar do the difference in the lipophilicity values for the diastereomers **1** and **2** (see Figure 7: Δlog*P* = 0.18 ~ 1.0 kJ mol^−1^). In contrast, the lipophilicity in **3** and **4** is the same (see Figure 7), indicating that their solvation by water is also close to identical. Therefore, no difference was found in the solvation effects contributing to the rotation barriers in these molecules.

**Figure 9:**
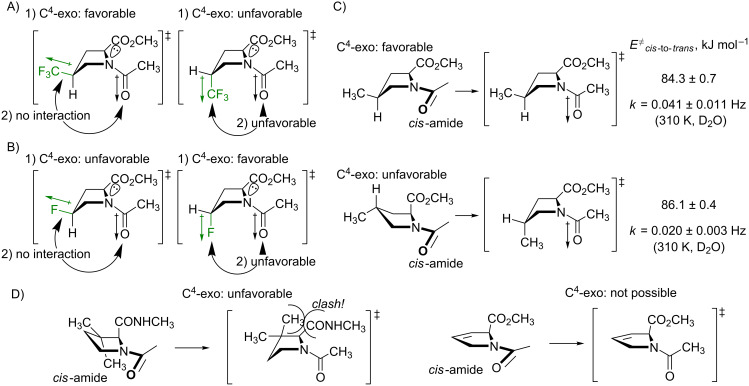
The explanation for the difference in the rotation barriers in the diastereomeric (A), 4-(trifluoromethyl)prolines (B) 4-fluoroprolines, and (**C)** 4-methylprolines. D) The two known cases with the increase of the barrier of rotation in proline analogues.

Finally, the difference between the barriers in the diastereomers calls for an explanation. It is clear from the presented data that the stereochemistry at the C^4^-atom has an effect on the rotation velocity. The difference was especially prominent in the trifluoromethylated species, **3** and **4** (see Table 3). Curiously, it is not possible to explain this finding by considering the ground state, thus the so-called *syn/exo* transition state [96] should be analyzed instead. The data make sense, if one considers that the transition state proceeds through the C^4^-*exo* conformation, as shown in Figure 9A. In this scenario, the *cis*-diastereomer **3** 1) readily favors the needed conformational state (vide supra), at the same time, 2) the CF_3_-group dipole is oriented perpendicular to the carbonyl-group dipole, and creates no repulsion. As the result, the transition state is energetically favored, and the rotation becomes faster. Conversely, in the other diastereomer **4**, both factors disfavor the transition state: 1) the ring should adopt the disfavored C^4^-*exo* conformation with an axial CF_3_ group, and 2) this group forms an unfavorable dipole–dipole interaction with the carbonyl group. As the result, the rotation process slows down. Eventually, these differences translate into the observed 2.0–2.6 kJ mol^−1^ energetic difference in the barriers.

The same considerations can be applied to the fluoroprolines, **1** and **2** (Figure 9B). However, in this case, the two arguments, 1) and 2) contradict each other. As a result, it was observed that the rotation in **2** is faster, but only by a small number: 0.4 and 0.5 kJ mol^−1^ in dichloromethane and benzene, respectively [97] (note that in water the ground-state solvation effect increases this difference).

The explanation proposed in Figure 9 relies on the assumption that the C^4^-*exo* conformation is favored by the transition state. This hypothesis should be further examined. This can be done by considering the rotation barriers in diastereomeric 4-methylprolines. In these species, the methyl group does not introduce any new dipoles to the parent system, therefore only the conformational preferences should have an effect. Indeed, it was found that the *cis*-to-*trans* transition in 4-*cis*-methylproline (favors C^4^-*exo*) is faster compared to 4-*trans*-methylproline (disfavors C^4^-*exo*) by 1.8 kJ mol^−1^ (Figure 9C). This observation agrees with the calculation predictions [98], and it fully confirms the hypothesis.

It is interesting to note, that there are only two types of analogues reported to date, where the barriers of the amide-bond rotation are higher compared to proline. These are 3,3-dimethylprolines [99] and 3,4-dehydroprolines [72]. It has not been clear however, why these analogues show such an effect, considering that the modifications are quite distant to the rotating amide bond. The inability of the analogues to adopt the C^4^-*exo* conformation in the transition state would explain the energy penalty in the rotation barriers (Figure 9D). In 3,3-dimethylprolines, the isomerization into the C^4^-*exo* envelope would lead to a steric clash between one of the methyl groups and the backbone carbonyl (*k* decreases by a factor of 2–6) [99]; in 3,4-dehydroprolines, the ring is unable to adopt an envelope conformation due to an endocyclic double bond (*k* decreases by factor 3) [72].

Overall, the conclusions regarding the ring conformation preference in the transition state provides an entirely new insight onto the peptide-bond isomerization, and can be helpful for better understanding of this fundamental process in natural and engineered systems.

### Effects in difluorinated spirocyclic analogues

**Figure 10:**
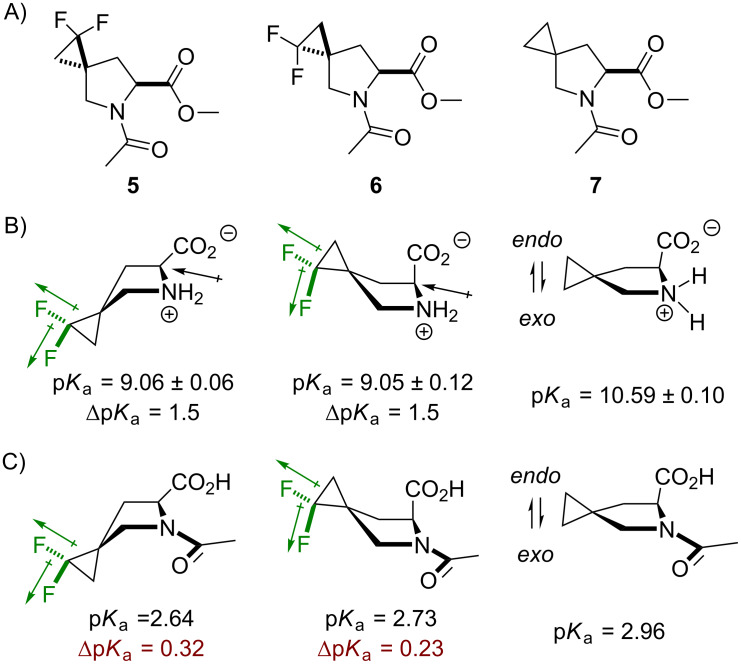
A) The structures of difluorinated model compounds **5** and **6**, and the fluorine-free reference **7**. B) Basicity data for the corresponding amino acids. C) Acidity data for the s-*cis N*-acetylamino acids. The error is ± 0.05.

In the final step, the proposed understanding of the molecular system was tested on a new set of proline analogues, **5** and **6** (Figure 10A). The synthesis of these amino acids was reported very recently [100]. The reference compound **7** was also analyzed. It should be noted that the difluorinated compounds **5** and **6** resemble the trifluoromethylated analogues **3** and **4** by the number of bonds separating the fluorine atoms from the main chain functional groups. A critical difference is, however, that the CF_2_ group present in **5** and **6** does not undergo a rotational averaging of the dipoles, as this happens in the C–CF_3_ system. This in principle may create even more complex situations compared to the presented so far.

In spite of this expectation, the results show that the model compounds **5** and **6** follow generally similar chiral bias to **3** and **4**. The p*K*_a_ data for the free amino acids shows that there is an identical reduction of the p*K*_a_ values from fluorination (Figure 10B and C). It was then found that neither the side-chain conformation markers (*J* coupling at 2-CH) nor the *trans/cis* equilibrium values (*K_trans/cis_*) were different in **5** compared to the non-fluorinated reference **7**. Likewise, the *trans/cis* equilibrium in **3** is similar to the unsubstituted proline. Conversely, in compound **6**, there was a higher content of the C^4^-*endo* conformation, a lower *trans/cis* equilibrium ratio, and the amide-bond rotation barrier was higher compared to the other diastereomer, **5**. The lack of rotational averaging in **5** and **6** leads to the interesting fact that the lipophilicity was different for these two diastereomers (Δlog*P* = 0.15). This suggests that the solvation by water slightly differs between the diastereomeric species. Indeed, this conclusion was further corroborated by the rotation barrier data, which showed higher Δ_water/benzene_ for the less lipophilic diastereomer, **6** (Table 4).

**Table 4:** Experimental data for compounds **5**–**7**.

compound	^1^H NMR multiplicity of 2-CH^a,b^						*E*^≠^*_cis_*_-to-_*_trans_*^c^ kJ mol^−1^		log*P*
	in CD_2_Cl_2_			in D_2_O			in D_2_O	in C_6_D_6_	
	^1^*J*, Hz	^2^*J*, Hz	C^4^-	^1^*J*, Hz	^2^*J*, Hz	C^4^-			

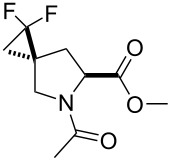 **5**	*K_trans/cis_* = 3.13 ± 0.07			*K_trans/cis_* = 3.94 ± 0.03			82.4 ± 0.4	73.4 ± 0.1	+0.18 ± 0.03
	8.8 (8.7)	5.0 (2.8)	mix^d^ *(endo)*	8.8 (8.8)	5.4 (2.3)	mix^d^ *(endo)*	Δ_water/benzene_ = 9.0		

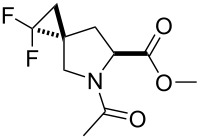 **6**	*K_trans/cis_* = 2.47 ± 0.02			*K_trans/cis_* = 3.15 ± 0.05			84.5 ± 0.2	74.2 ± 0.2	+0.03 ± 0.02
	8.9 (8.5)	1.8 (1.0)	*endo* *(endo)*	9.1 (8.6)	1.2 (n.d.)^e^	*endo* *(endo)*	Δ_water/benzene_ = 10.3		

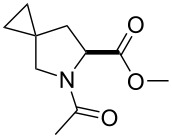 **7**	*K_trans/cis_* = 3.35 ± 0.02			*K_trans/cis_* = 3.92 ± 0.04			85.6 ± 1.1	76.3 ± 0.1	+0.19 ± 0.03
	8.6 (8.5)	4.4 (2.4)	mix^d^ *(endo)*	8.8 (8.4)	3.6 (n.d.)^e^	mix^d^ *(endo)*	Δ_water/benzene_ = 9.5		

^a^Read out from 1D ^1^H NMR spectra recorded at 700 MHz frequency at 298 K; ^b^the s-*trans* (major) amide rotamer data is shown first; the results in parentheses are for the s-*cis* (minor) amide rotamer; ^c^measured by ^1^H EXSY NMR at 298 K for C_6_D_6_ and 310 K for D_2_O; ^d^mix = a mixture of two conformations; ^e^n.d. not detected.

Overall, the effects from fluorination in both diastereomeric compounds, **5** and **6**, were found to be weak relative to what has been observed in monofluoroprolines, for instance. Nonetheless, the trends in the molecular properties come in a good agreement with analogous trends found in (trifluoromethyl)prolines, with some exceptions in lipophilicity and solvation.

## Conclusion

In summary, this study aimed to expose the impact of polar substituents in position 4 of the proline ring onto the physicochemical properties of the parent amino acid residue. Two molecular systems were examined to mimic such situation: 4-fluoroproline and 4-(trifluoromethyl)proline. In the first one, the substituent C^4^–F bond tends to adopt an axial position, due to a F–C–C–N *gauche* effect. In the second one, the C^4^–CF_3_ bond favors an equatorial position due to its large steric size. The resulting orientation of the substituent dipoles with respect to the main chain groups has an impact onto molecular properties such as lipophilicity, acid–base transition, and kinetics of the amide-bond rotation. Interestingly it was found that the side-chain conformational preferences translate differently into the energy of the *trans/cis* amide equilibrium. While in the monofluoroprolines the effect was relatively strong, in the trifluoromethylated species it was notably weaker. Thus, it is expected that (trifluoromethyl)prolines should impact polypeptide structures by altering the bulkiness and hydrophobicity of the residue site, and not the backbone folding. The conclusions derived from the trifluoromethylated species were further corroborated by the examination of 1,1-difluoro-5-azaspiro[2.4]heptane-6-carboxylate based models, which showed similar trends in properties.

Finally, the study provides a set of experimental evidences that reveal details of the transition state for the *cis/trans* amide rotation. Namely, it follows from the presented data that the transition state prefers the C^4^-*exo* conformation of the proline ring. This creates a chiral bias of the C^4^-substitution in the amide-rotation barrier, that has not been highlighted so far in the experimental literature. These findings will be helpful for the design and the use of proline analogues in complex biological systems such as peptides and proteins, especially in ^19^F NMR labelling, where fluorinated prolines can serve as spectroscopic probes. Potential areas for the application of fluorinated prolines are numerous, and include the design of molecular recognition systems [101], organocatalysis [102], drug discovery [103] and more.

## Supporting Information

File 1 Data on acid–base transition and amide bond isomerism and NMR characterization of compounds **1**–**7**.
