# Supplementary material for: Polarity effects in 4-fluoro- and 4-(trifluoromethyl)prolines
Source: Beilstein J Org Chem. 2020 Jul 23;16:1837–52. doi: 10.3762/bjoc.16.151 (PMC7385359; doi:10.3762/bjoc.16.151)
Supplement: File 1 — Data on acid–base transition and amide bond isomerism and NMR characterization of compounds 1–7. [file Beilstein_J_Org_Chem-16-1837-s001.pdf]

BEILSTEIN JOURNAL OF ORGANIC CHEMISTRY

Supporting Information

for

Polarity effects in 4-fluoro- and 4-(trifluoromethyl)prolines

Vladimir Kubyshkin

Beilstein J. Org. Chem. **2020,** *16,* 1837–1852. doi:10.3762/bjoc.16.151

Data on acid–base transition and amide bond isomerism and NMR characterization of compounds 1–7

License and Terms: This is a supporting information file under the terms of the Creative Commons Attribution License (http://creativecommons.org/licenses/by/4.0). Please note that the reuse, redistribution and reproduction in particular requires that the authors and source are credited.
The license is subject to the *Beilstein Journal of Organic Chemistry* terms and conditions: (https://www.beilstein-journals.org/bjoc)

Index

Experimental description S2

Sample preparation S2

NMR measurements S3

EXSY procedure description S4

Molecular modeling S8

Figure S1 .... S8

Extended physicochemical data S9

NMR data for compounds **1–7** S13

References S30

Experimental description

Sample preparation

Pure enantiomeric (2*S*,4*S*)- and (2*S*,4*R*)-fluoroprolines were obtained from commercial sources and processed as described.[S1] The methyl ester of *N*-Boc-(2*S*,4*S*)-trifluoromethylproline was obtained as described.[S2] Its partial epimerization at position 2 was received in an attempt to saponificate the material with an excess of 1 M NaOH in methanol under overexposure. The non-converted methyl ester was recovered in about 0.33 g amount with the ratio of the (2*S*,4*S*) and (2*R*,4*S*) isomers of about 3:2. This material was processed towards the model compounds **3**, and **4** that were eventually separated on a silica gel column (ethyl acetate/methanol 40:1 to 20:1, **4** eluted first). Commercial (5-(*tert*-butoxycarbonyl)-1,1-difluoro-5-azaspiro[2.4]heptane-6-carboxylic acid was purchased from a commercial supplier. The ratio of the diastereomers (3*R*,6*S*):(3*R*,6*R*) was about 2:1 according to the report of their synthesis from the company.[S3] The diastereomers were separated using preparative reversed phase HPLC (C18 column) with water/acetonitrile gradient elution (no ion-pairing additives). Resulting species were enriched to about 80% of pure diastereomers. Their further assignment was achieved by $^{1}H^{19}F$ HOESY experiments (mixing time 500 ms). The compounds were processed towards the model compounds **5** and **6**. Final purification on a silica gel column (ethyl acetate/methanol 40:1 to 20:1) allowed complete separation of the diastereomers in pure forms, while compound **5** eluted first. Finally, (*6S*)-(5-(*tert*-butoxycarbonyl)-5-azaspiro[2.4]heptane-6-carboxylic acid was obtained from commercial sources.

The synthesis of the model compounds **3–7** was performed on 0.2–0.1 g scales using the following general schemes:

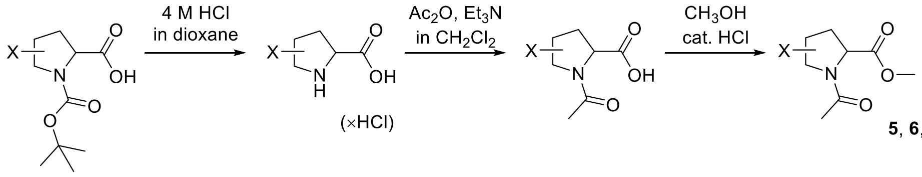

5, **6**, **7**

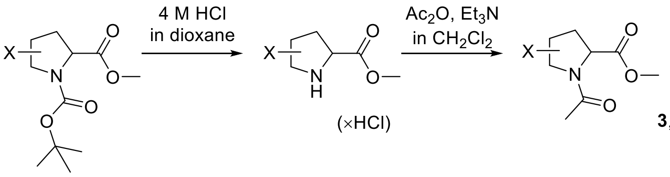

3, **4**

All manipulations were performed at room temperature under air atmosphere. An *N*-Boc amino acid was treated with an excess of 4 M hydrogen chloride in dioxane for 90–120 min. The solvent was removed under reduced pressure, the residue dissolved in water and freeze-dried. To the residue anhydrous dichloromethane was added, followed by acetic anhydride (2–5 equiv), and trimethylamine (2–5 equiv). The resulting mixture was stirred for about 1 hour. The solvent was removed under reduced pressure, the residue dissolved in water and freeze-dried. The resulting mixture was dissolved in water and filtered through a short cation exchange column. The acidic fractions were collected and freeze-dried. The *N*-acetylated compound obtained in this way was dissolved in methanol (≈ 1–2 mL) and trimethylsilane (≈ 0.1–0.2 mL) was added to acidify the mixture. The mixture was stirred for several hours, the solvent was removed under reduced

pressure, and the residue purified on a short silica gel column using ethyl acetate/methanol 40:1 to 20:1 gradient elution. The compounds **3–7** were obtained as colorless oils.

NMR experiments

NMR experiments were performed with spectrometers operating at 500 and 700 MHz proton frequency. The variable temperature unit was calibrated according to conventional methanol standard. The pK_a values were obtained from analytical samples according to the detailed protocols described earlier.[S1,S4] The samples were titrated at 295–298 K, and the measurements were performed at 298 K. The lipophilicity measurements were performed as described.[S1] A compound (4–6 mg) was shaken with deionized water and octan-1-ol (1.00 mL each) for about 24 hours at 295–298 K. Then, 0.30 mL of each phase were taken in NMR tubes and diluted with 0.30 mL of acetonitrile-d_3. The measurements were performed with either ^{1}H or ^{19}F detection using datasets with identical settings. The equilibrium ratio (P) was determined as the ratio between absolute integral values from the octanol and water samples. The measurements were performed in triplicate.

For the amide isomerism measurements 4–6 mg of an analyte (**1–7**) was dissolved in a solvent (0.53 mL) in a standard 5 mm NMR tube. The ^{1}H and ^{19}F NMR measurements were performed manually in one scan in order to enable complete prerelaxation of the nuclei. The ratios between equivalent integrals originating from rotameric species were taken as the equilibrium constants (K). The value was averaged from few different resonances, few different spectra, ^{1}H and ^{19}F measurements.

The rotation velocities were measured in 2D cross-relaxation pulse sequences with z gradients: ^{1}H EXSY (for all samples), ^{19}F EXSY (for **3** and **4**) and ^{19}F{^{1}H} EXSY with proton decoupling during evolution, mixing and detection (for **1** and **2**). The measurements in aqueous samples (D_2O) were performed at 310 K, the measurements in benzene (C_6D_6), and dichloromethane (CD_2Cl_2) were performed at 298 K. The frequency domains were zoomed to the regions of interest, and the experiments were performed with standard settings and prolonged recycling delays ($\geq 3 \cdot t_1$ of the analyzed resonances). The frequency domain spectra were baseline corrected and integrated. The exchange rate matrices were calculated using EXSYCalc freeware (Mestrec). A detailed description is provided below.

EXSY procedure description

For the compound/solvent combination **4**/C_6D_6, first the $K_{trans/cis}$ was identified from the 1D ^{1}H and ^{19}F NMR spectra as 4.69 ± 0.14:

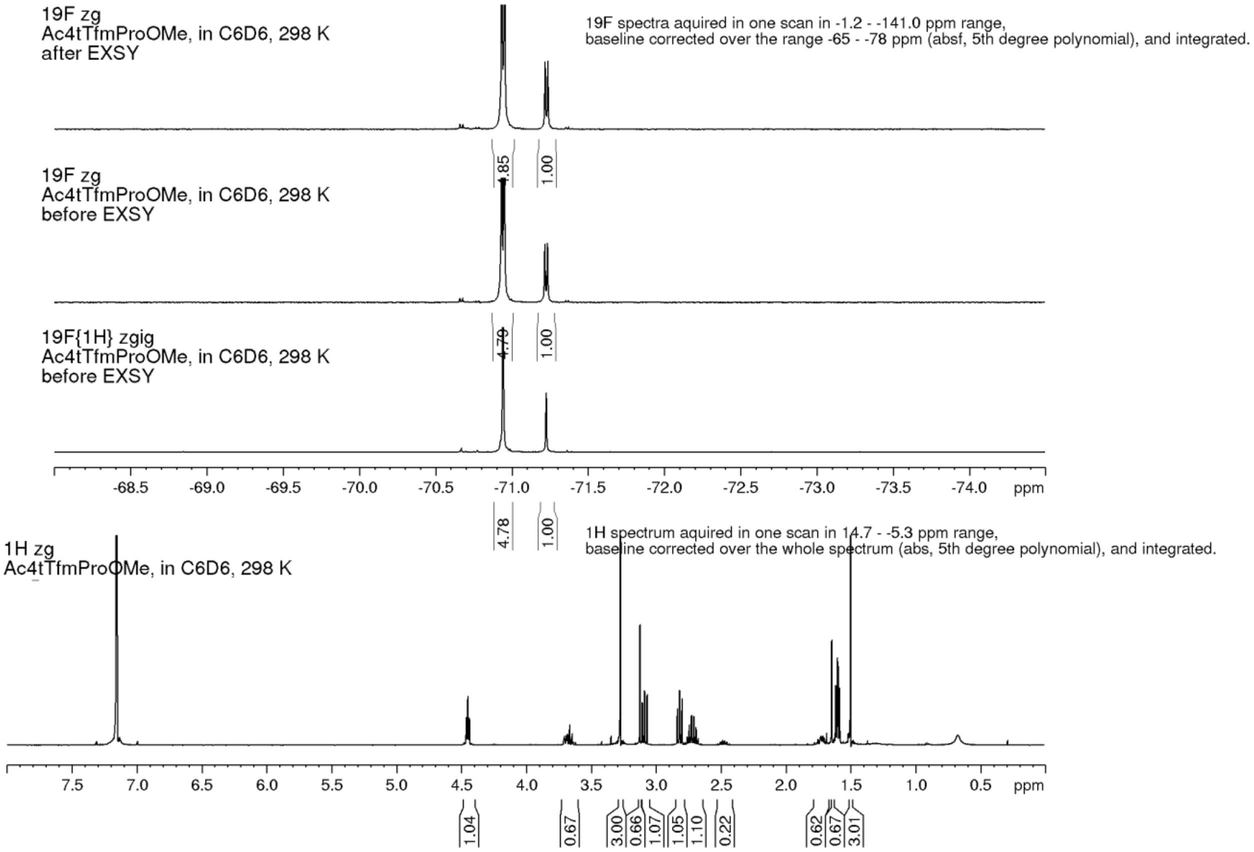

Then, ^{1}H EXSY were acquired with mixing times 0.5 and 1 s, with the parameters as outlined below:

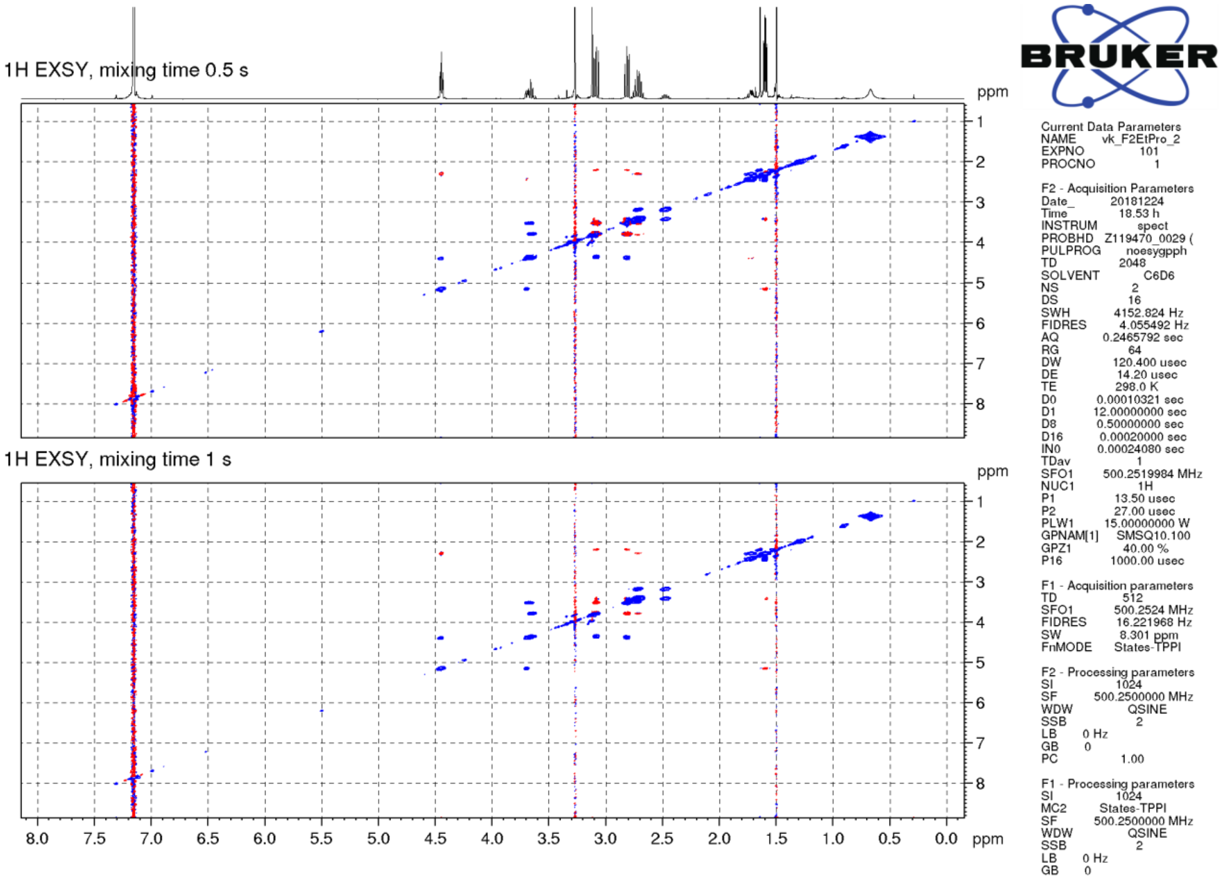

Note prolonged recycling delay (d1). According to our experience, a prolonged recycling delay does not change the final exchange rate, but it substantially improves the agreement between the rate constants derived from analysis of individual resonances. Effectively, this helps to reduce the standard deviation in the final value.

There is also a relatively high number of data points in the indirect dimension (TD1 512). This enables suppression of truncation artifacts in the indirect dimension, and effectively the sharp CH_3-group resonances could be integrated and analyzed.

^{19}F EXSY spectra were acquired with mixing times 0.3, 0.6, and 0.9 s with the parameters as shown below:

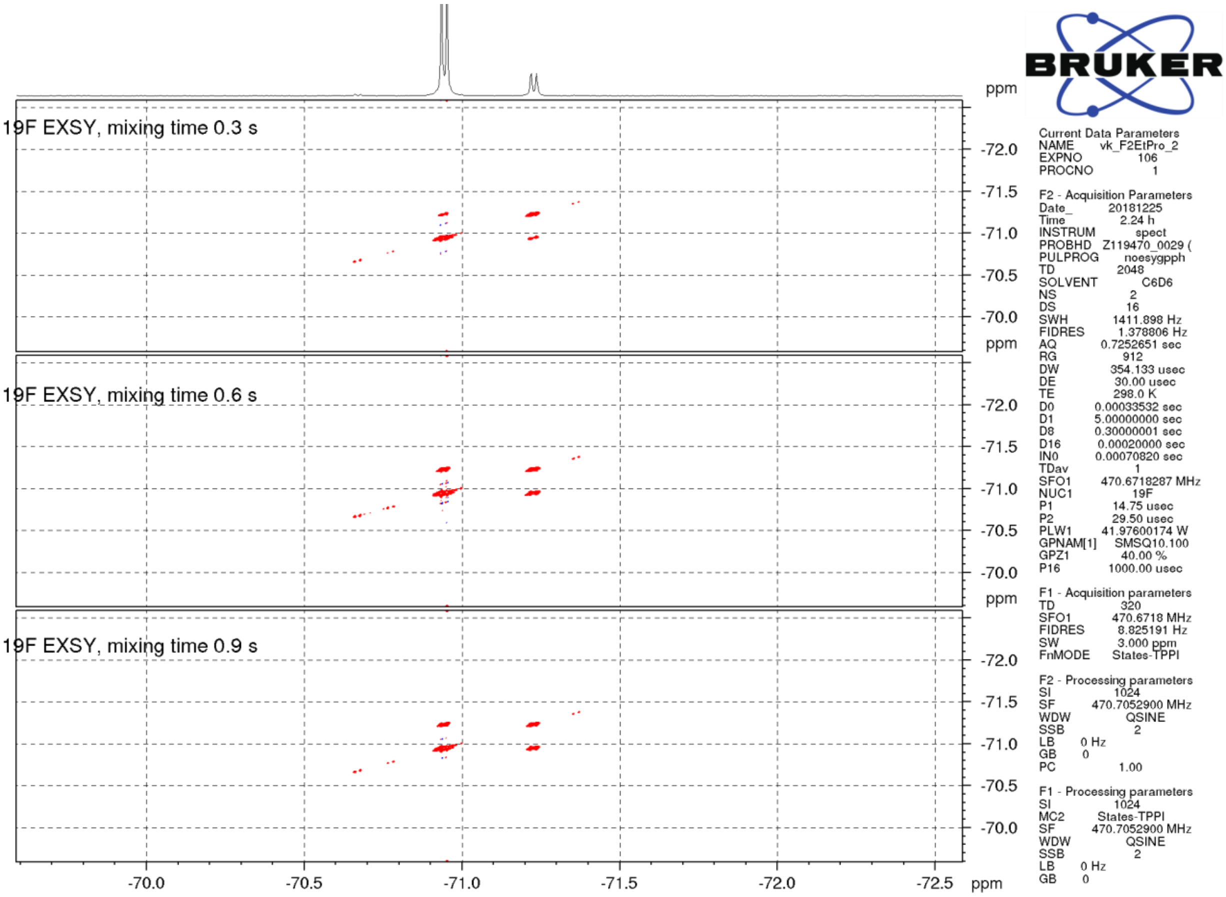

Here, prolonged recycling was also applied. The t_1 value was estimated as ≈ 1.4 s according to an inversion recovery experiment. Hence, the spin recycling aq+d1 = 5.7 s ≥ 4·t_1. Interestingly, the inversion recovery showed that the t_1 for the s-*cis* resonance is slightly longer.

The frequency domain spectra were phased in direct and baseline corrected in both dimensions. In the ^{1}H EXSY, the CH_3O and acetyl CH_3 resonances were integrated. In the ^{19}F EXSY, the fluorine resonance was integrated. Relative integrals were input into EXSYcalc as shown:

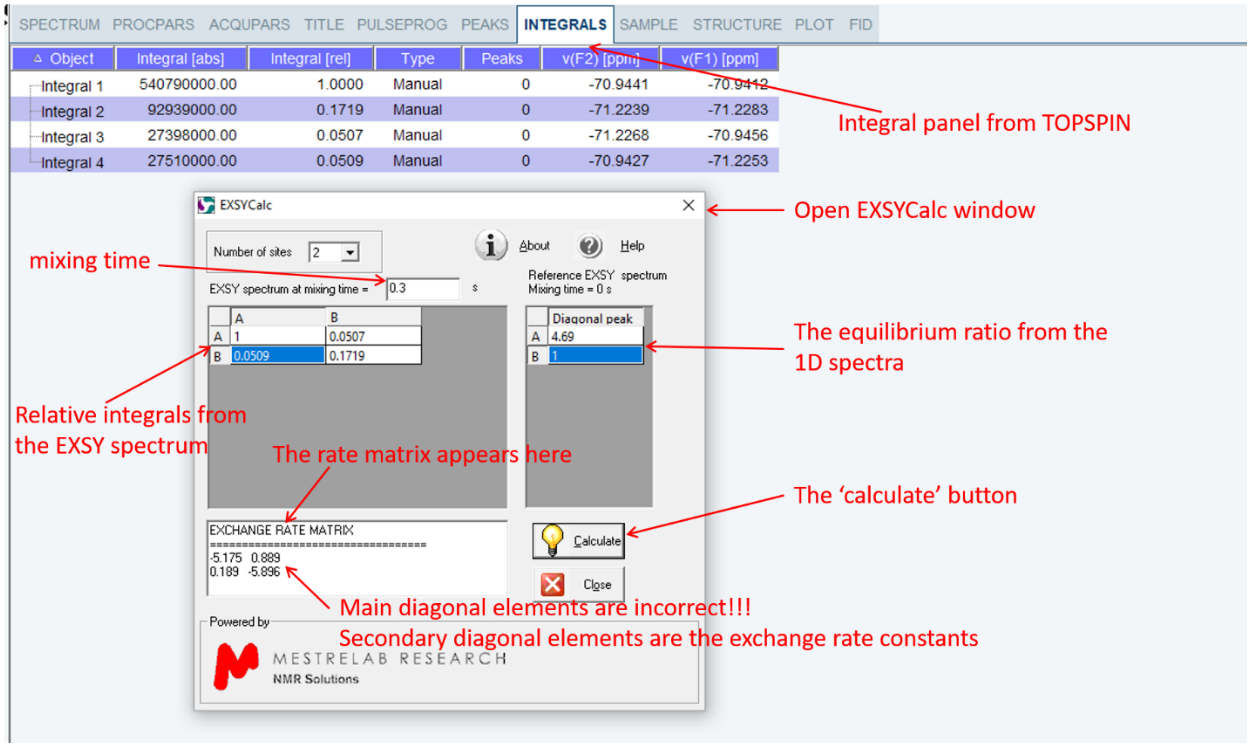

The exchange rate matrix is defined as:

$$\begin{bmatrix} -R_1 - k_1 & k_{-1} \\ k_1 & -R_2 - k_{-1} \end{bmatrix}$$

The main diagonal elements contain the relaxation terms (R_1 and R_2) that are incorrect in the current treatment. To obtain a fully correct exchange rate matrix, a reference EXSY spectrum with zero mixing time should be acquired, and absolute integral values should be inserted into the EXSYCalc. With the existed method, only the secondary diagonal elements that represent the exchange rates are correct.

In the current example (**4**/C_6D_6), the following exchange rates were obtained:

	^{1}H EXSY				^{19}F EXSY		
	mix 0.5 s		mix 1 s		mix 0.3 s	mix 0.6 s	mix 0.9 s
	CH_3O	Ac	CH_3O	Ac			
k_{-1}, Hz	0.829	0.872	0.823	0.811	0.889	0.879	0.886
k_1, Hz	0.182	0.175	0.179	0.170	0.189	0.190	0.188

The rates were averaged to deliver the final value that is presented in Table S2. The standard deviation values were also calculated from the measured values.

Results: $k_{trans\text{-}to\text{-}cis}$ = 0.182 ± 0.007 Hz; $k_{cis\text{-}to\text{-}trans}$ = 0.855 ± 0.031 Hz

Molecular modeling

The molecular modeling was performed with compounds **1–4** using Scigress Modelling Suite (Fujitsu, Poland). Dipole calculations were performed after geometry optimization using the PM6 Hamiltonian included in the MOPAC package.

The C^4-*exo/endo* conformations were extracted from dynamic simulations of potential energy map generated in MM3 force field. The dihedral angles H–C^2–C^3–H were estimated from resulting conformations. The experimental $^2J_{HCCH}$ were then converted to dihedral angles using MestReJ freeware (Mestrec) with the following substitutes: S1=NRC(=O)R(s-cis), S2=COOR, S3,S4=H/CHClMe as the closest ones from the existing list. The conformations were readjusted to fit both dihedral angles within ± 10° accuracy. The ranges for the dihedral angles in the C^4-*endo* and C^4-*exo* conformations were estimated from the ones obtained in the simulation run before and after the adjustments. The estimated dihedral angle ranges were then converted to the *J* value ranges. These are shown in Figure 4.

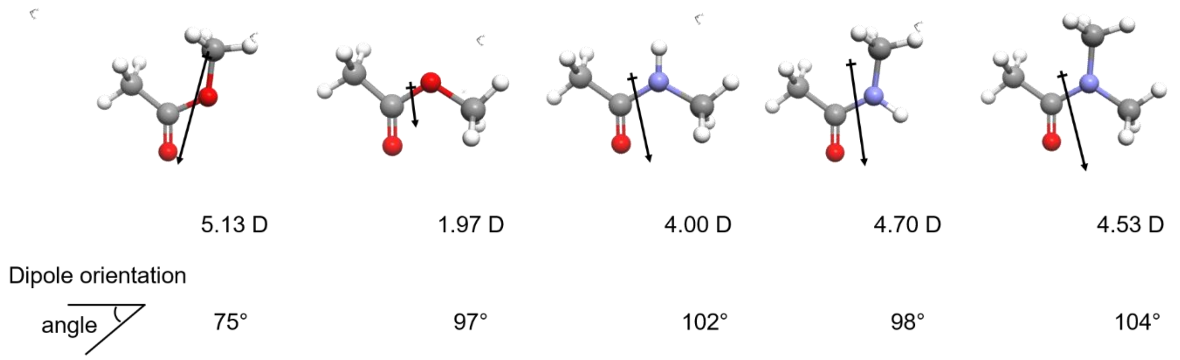

Figure S1 Dipole size and orientation in methyl acetate and *N*-methyl- and *N*,*N*-dimethylacetamides. The simulations were done using PM6 Hamiltonian from MOPAC package.

Extended physicochemical data

Table S1 Acid–base transition

compound	pK_a	compound	X = OH				X = O^-	ΔpK_a*
			rotamer		ΔpK_a	$K_{trans/cis}$	$K_{trans/cis}$	
			s-*cis*	s-*trans*				
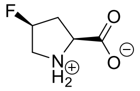	9.10±0.10	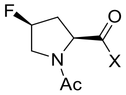	2.87	3.39	0.52	2.27±0.02	0.68±0.04	0.52
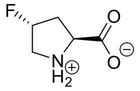	9.10±0.10	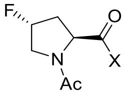	2.37	3.19	0.82	5.30±0.20	0.91±0.02	0.77
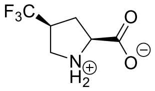	8.42±0.05	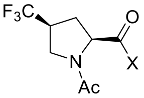	2.60	3.18	0.58	3.53±0.06	0.89±0.02	0.60
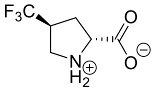	8.43±0.05	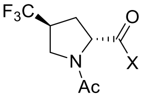	2.55	3.15	0.60	2.86±0.04	0.75±0.02	0.58
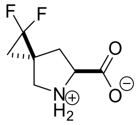	9.06±0.06	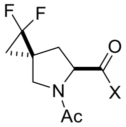	2.64	3.18	0.54	3.13±0.09	0.76±0.01	0.61
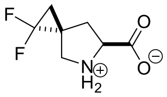	9.05±0.12	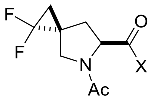	2.73	3.30	0.57	2.65±0.10	0.67±0.02	0.60
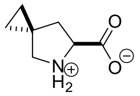	10.59±0.10	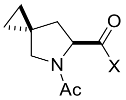	2.96	3.57	0.61	3.14±0.15	0.80±0.01	0.59

The ΔpK_a values are obtained according to the equation:

$$\Delta pK_a = pK_a(s - trans) - pK_a(s - cis)$$

The ΔpK_a* values are obtained according to the equation[S4]:

$$\Delta pK_a^* = log_{10}\frac{K_{trans/cis}(X = OH)}{K_{trans/cis}(X = O^-)}$$

Thus, the first value is obtained from the titration experiments, while the second is obtained from equilibrium measurements at extreme pH. In principle, both values (columns highlighted in yellow) should be equal within the accuracy of the experimental determination.

The values for fluoroproline derivatives are from reference.[S4]

Table S2 Amide bond isomerism

compound	$K_{trans/cis}$			k, Hz						$E^{\neq}$, kJ mol^{-1}					
				cis-to-*trans*			*trans*-to-*cis*			*cis*-to-*trans*			*trans*-to-*cis*		
	in C_6D_6	in CD_2Cl_2	in D_2O	in C_6D_6	in CD_2Cl_2	in D_2O	in C_6D_6	in CD_2Cl_2	in D_2O	in C_6D_6	in CD_2Cl_2	in D_2O	in C_6D_6	in CD_2Cl_2	in D_2O
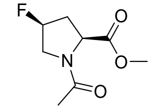	2.09±0.03	1.71±0.07	2.57±0.08	0.463±0.034	0.187±0.003	0.041±0.004	0.223±0.008	0.109±0.002	0.016±0.002	74.9±0.2	77.1±0.1	84.3±0.2	76.6±0.1	78.5±0.1	86.7±0.3
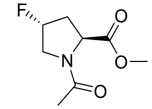	5.38±0.22	4.64±0.10	7.08±0.27	0.556±0.027	0.220±0.016	0.087±0.009	0.103±0.004	0.049±0.005	0.012±0.002	74.4±0.2	76.7±0.2	82.3±0.3	78.6±0.1	80.5±0.2	87.4±0.5
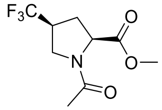	5.39±0.09	3.22±0.06	4.36±0.11	2.44±0.07	0.690±0.019	0.131±0.008	0.453±0.011	0.211±0.003	0.031±0.002	70.8±0.1	73.9±0.1	81.3±0.1	74.9±0.1	76.8±0.2	85.0±0.1
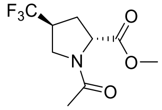	4.69±0.14	2.66±0.11	3.57±0.13	0.855±0.031	0.265±0.007	0.060±0.006	0.182±0.007	0.098±0.009	0.018±0.002	73.4±0.1	76.3±0.1	83.3±0.3	77.2±0.1	78.7±0.3	86.4±0.3
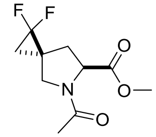	4.05±0.03	3.13±0.07	3.94±0.03	0.854±0.025	0.287±0.007	0.085±0.011	0.209±0.004	0.093±0.002	0.020±0.001	73.4±0.1	76.1±0.1	82.4±0.4	76.9±0.1	78.9±0.1	86.1±0.1
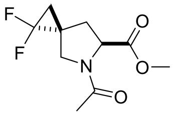	3.23±0.05	2.47±0.02	3.15±0.05	0.620±0.025	0.188±0.007	0.038±0.004	0.189±0.003	0.074±0.003	0.011±0.002	74.2±0.1	77.1±0.1	84.5±0.2	77.1±0.1	79.4±0.1	87.6±0.6
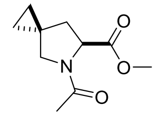	4.13±0.04	3.35±0.02	3.92±0.04	0.264±0.010	0.080±0.004	0.024±0.008	0.062±0.002	0.023±0.002	0.006±0.001	76.3±0.1	79.2±0.2	85.6±1.1	79.9±0.1	82.3±0.3	89.2±0.5

The equilibrium $K_{trans/cis}$ values were determined at 298 K. The rotation velocities k were determined at 298 K for benzene and dichloromethane solutions, and at 310 K for aqueous solutions. The activation energies were calculated using the Eyring equation:

$$E^{\neq} = -RT\left(ln\frac{k}{T} - 23.76\right),$$

where R – gas constant, T – absolute temperature. The standard deviation takes into account discrepancies between the measurements (different mixing times, ^{1}H, ^{19}F detection), and do not take into account the temperature error.

For 4-methylproline derivatives[S1] the rotation velocities were determined in deuterium oxide buffered by a phosphate buffer to pH 7, at 310 K:

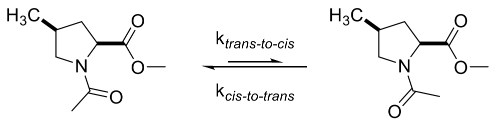

$K_{trans/cis}$ (298 K) = 8.05 ± 0.13

$k_{cis\text{-}to\text{-}trans}$ = 0.041 ± 0.011 $E^{\neq}$ = 84.3 ± 0.7 kJ mol^{-1}

$k_{trans\text{-}to\text{-}cis}$ = 0.004 ± 0.001 $E^{\neq}$ = 90.3 ± 0.7 kJ mol^{-1}

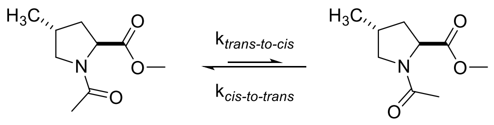

$K_{trans/cis}$ (298 K) = 4.67 ± 0.14

$k_{cis\text{-}to\text{-}trans}$ = 0.020 ± 0.003 $E^{\neq}$ = 86.1 ± 0.4 kJ mol^{-1}

$k_{trans\text{-}to\text{-}cis}$ = 0.005 ± 0.001 $E^{\neq}$ = 89.7 ± 0.6 kJ mol^{-1}

Table S3 Chemical shifts for the 2-CH resonances in ^{1}H NMR spectra

compound	in D_2O		in CD_2Cl_2		in C_6D_6	
	s-*trans*	s-*cis*	s-*trans*	s-*cis*	s-*trans*	s-*cis*
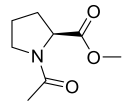	4.36	4.62	4.42	4.40	4.34	3.68
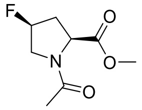	4.68	4.84	4.72	4.54	4.64	3.72
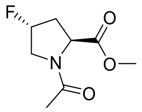	4.50	4.84	4.52	4.60	4.62	4.04
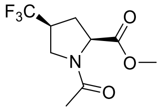	4.49	4.81	4.49	4.52	4.25	3.65
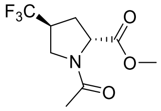	4.56	4.82	4.63	4.56	4.45	3.69
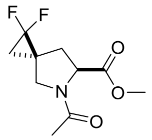	4.58	4.87	4.62	4.59	4.57	3.77
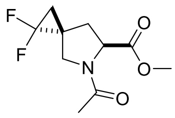	4.64	4.82	4.67	4.55	4.66	3.84
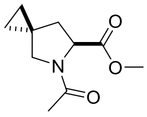	4.55	4.75	4.58	4.52	4.70	3.93

Note that the chemical shifts were read out from the ^{1}H NMR spectra acquired at 298 K. The spectra were referenced according to the conventional deuterium lock referencing (Bruker Avance III console, for solvent details see command 'edlock' in Topspin). No additional referencing was applied.

NMR data for compounds 1–7

For earlier characterization of compounds see: **1**, **2**[S1,S5,S6], **3**[S1,S2].

Methyl (*2S,4S*)-1-acetyl-4-fluoroprolinate (**1**)

^{1}H NMR (500 MHz, D_2O), two rotamers, $K_{trans/cis}$ = 2.57 ± 0.08

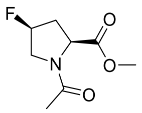

s-*trans*: 5.35 (dtd, *J* = 52, 3.2, 1.4 Hz, 1H, γ-CH), 4.67 (dd, *J* = 8.8, 2.9 Hz, 1H, α-CH), 3.91 (ddd, *J* = 25, 13.3, 1.1 Hz, 1H, δ-CH), 3.85 (ddd, *J* = 39, 13.1, 3.6 Hz, 1H, δ-CH), 3.70 (s, 3H, CH_3O), 2.46 (m, 2H, β-CH_2), 2.07 (s, 3H, Ac);

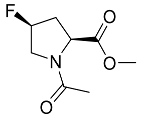

s-*cis*: 5.32 (dt, *J* = 52, 3.4 Hz, 1H, γ-CH), 4.84 (d, *J* = 9.6 Hz, 1H, α-CH), 3.74 (s, 3H, CH_3O), 3.70 (ddd, *J* = 28, 14.8, 1.6 Hz, 1H, δ-CH), 3.67 (ddd, *J* = 38, 14.5, 4.0 Hz, 1H, δ-CH), 2.64 (tm, *J* = 16.0 Hz, 1H, β-CH), 2.51 (m, 1H, β-CH), 2.00 (s, 3H, Ac).

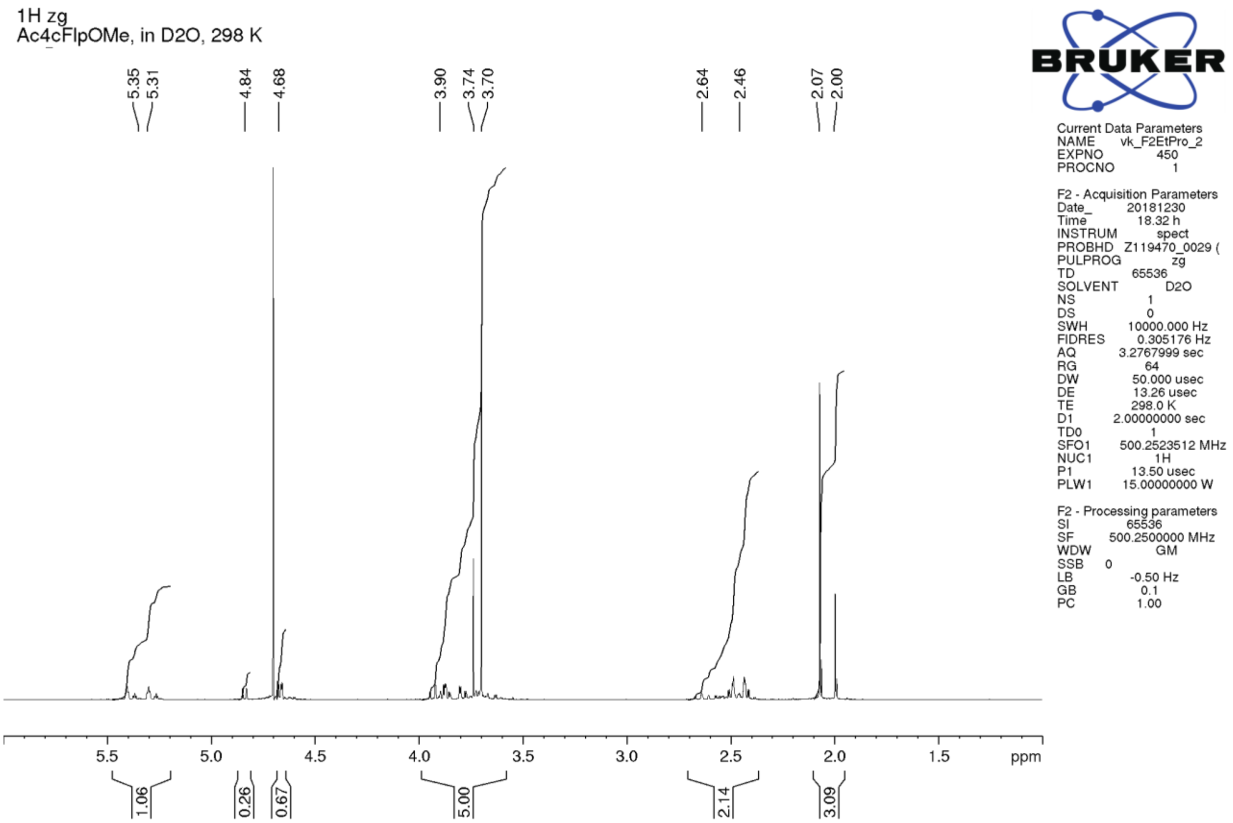

$^{19}F\{^{1}H\}$ NMR (471 MHz, D_2O):

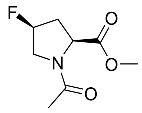

s-*trans*: −173.27 (s);

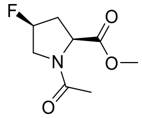

s-*cis*: −173.25 (s).

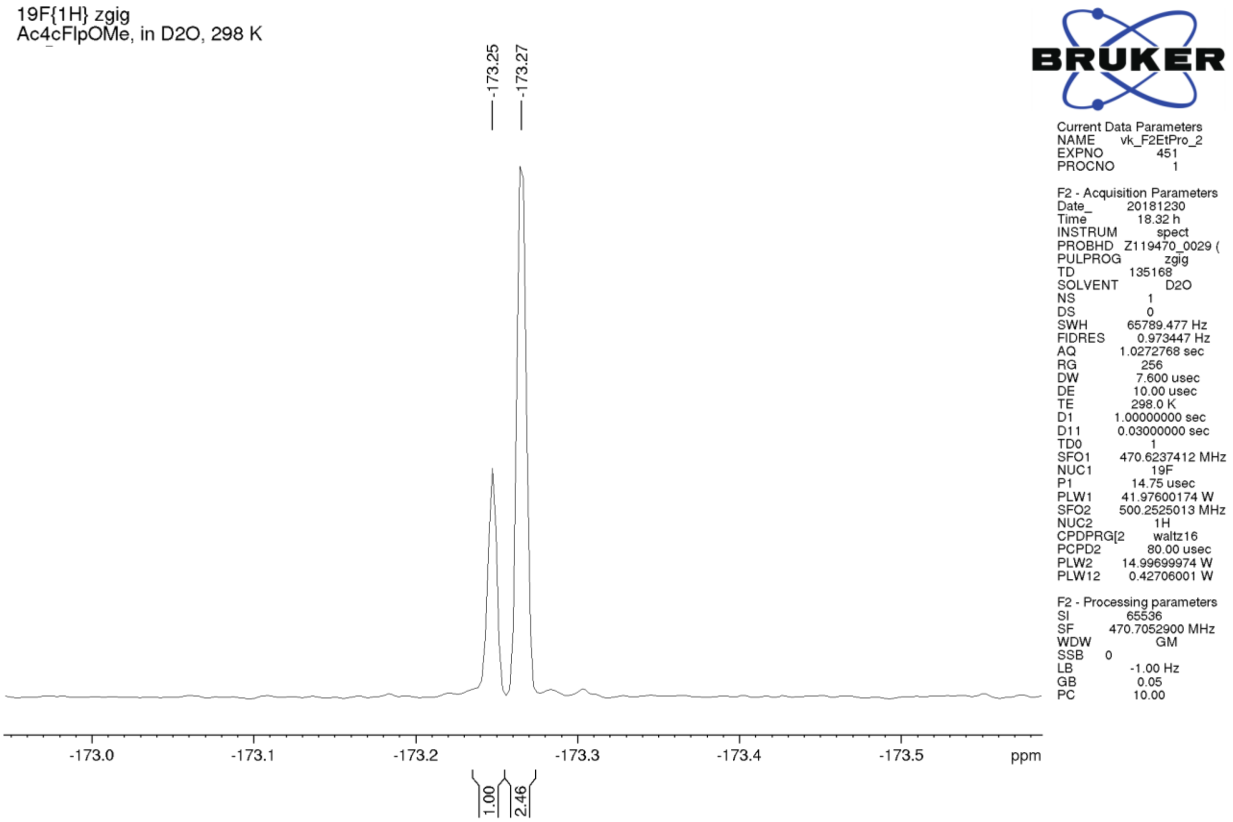

Methyl (*2S,4R*)-1-acetyl-4-fluoroprolinate (**2**)

^{1}H NMR (500 MHz, D_2O), two rotamers, $K_{trans/cis}$ = 7.08 ± 0.27

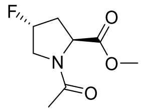

s-*trans*: 5.36 (dt, *J* = 52, 3.4 Hz, 1H, γ-CH), 4.50 (dd, *J* = 10.0, 7.8 Hz, 1H, α-CH), 3.94 (ddd, *J* = 22, 13.1, 2.3 Hz, 1H, δ-CH), 3.83 (ddd, *J* = 38, 13.4, 3.3 Hz, 1H, δ-CH), 3.70 (s, 3H, CH_3O), 2.63 (m, 1H, β-CH), 2.14 (dddd, J = 42, 14.2, 10.2, 3.8 Hz, 1H, β-CH), 2.06 (s, 3H, Ac);

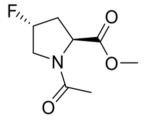

s-*cis*: 5.30 (dt, *J* = 52, 3.9 Hz, 1H, γ-CH), 4.84 (t, *J* = 8.4 Hz, 1H, α-CH), 4.03 (ddd, *J* = 21, 13.6, 2.6 Hz, 1H, δ-CH), 3.75 (s, 3H, CH_3O), 3.46 (ddd, *J* = 38, 14.3, 3.5 Hz, 1H, δ-CH), 2.77 (m, 1H, β-CH), 2.36 (m, 1H, β-CH), 1.95 (s, 3H, Ac).

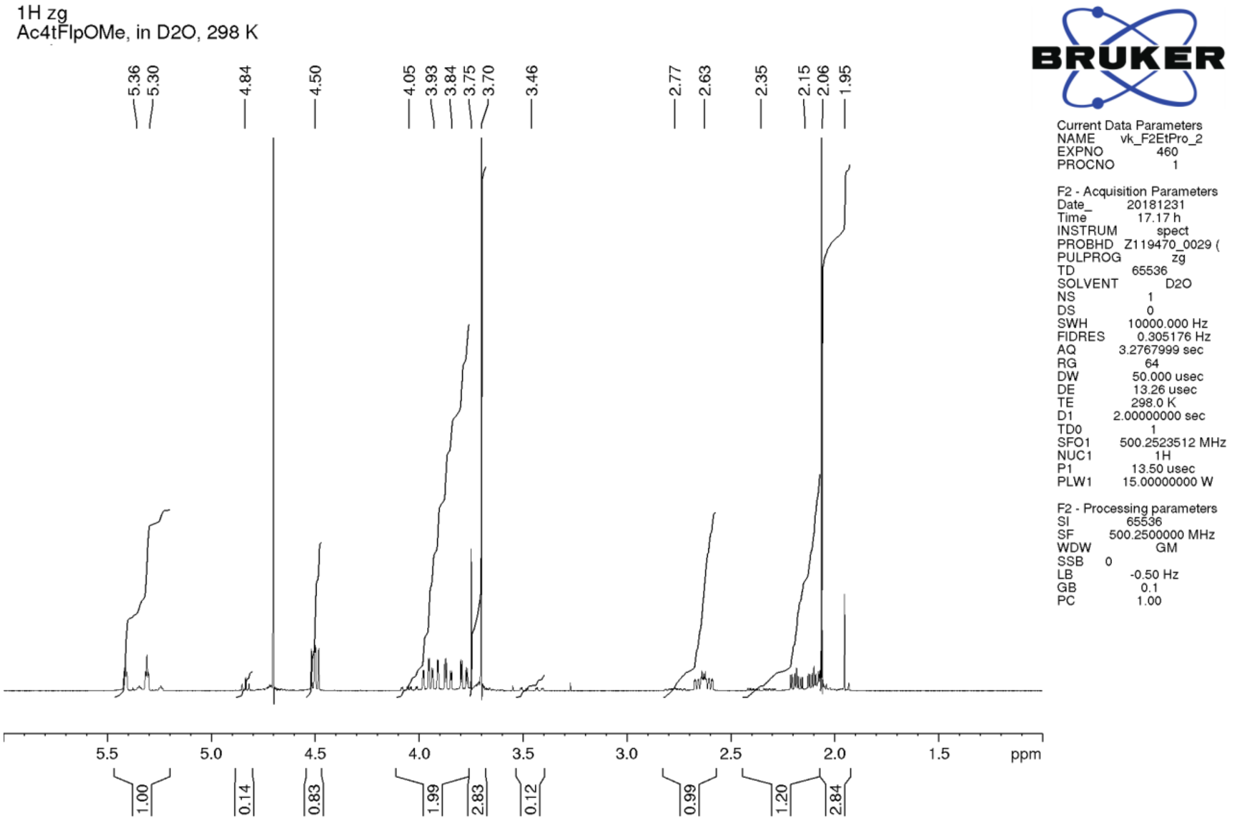

$^{19}F\{^1H\}$ NMR (471 MHz, D_2O):

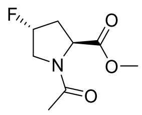

s-*trans*: −177.86 (s);

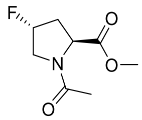

s-*cis*: −177.80 (s).

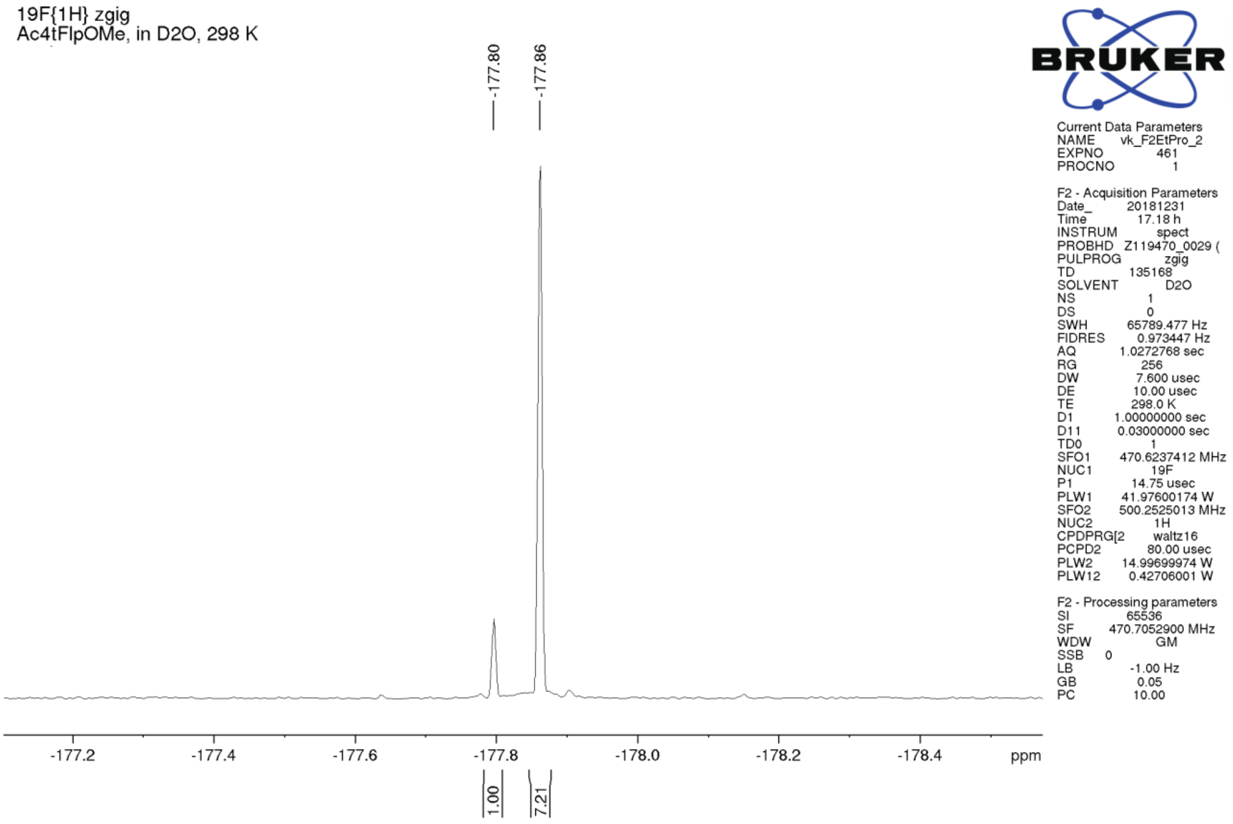

Methyl (*2S,4S*)-1-acetyl-4-trifluoromethylprolinate (**3**)

^{1}H NMR (500 MHz, D_2O), two rotamers, $K_{trans/cis}$ = 4.36 ± 0.11

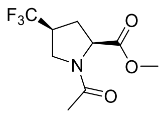

s-*trans*: 4.49 (t, *J* = 8.3 Hz, 1H, α-CH), 3.99 (dd, *J* = 11.0, 8.4 Hz, 1H, δ-CH), 3.70 (s, 3H, CH_3O), 3.69 (dd, *J* = 11.0, 9.1 Hz, 1H, δ-CH), 3.27 (m, 1H, γ-CH), 2.62 (dt, *J* = 13.4, 8.4 Hz, 1H, β-CH), 2.13 (dt, *J* = 13.4, 8.4 Hz, 1H, β-CH), 2.06 (s, 3H, Ac);

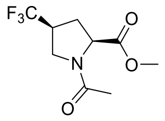

s-*cis*: 4.81 (dd, *J* = 9.9, 4.1 Hz, 1H, α-CH), 3.97 (m, 1H, δ-CH), 3.74 (s, 3H, CH_3O), 3.43 (dd, *J* = 12.8, 6.4 Hz, 1H, δ-CH), 3.16 (m, 1H, γ-CH), 2.74 (dt, *J* = 14.5, 9.5 Hz, 1H, β-CH), 2.43 (dt, *J* = 14.3, 4.5 Hz, 1H, β-CH), 1.96 (s, 3H, Ac).

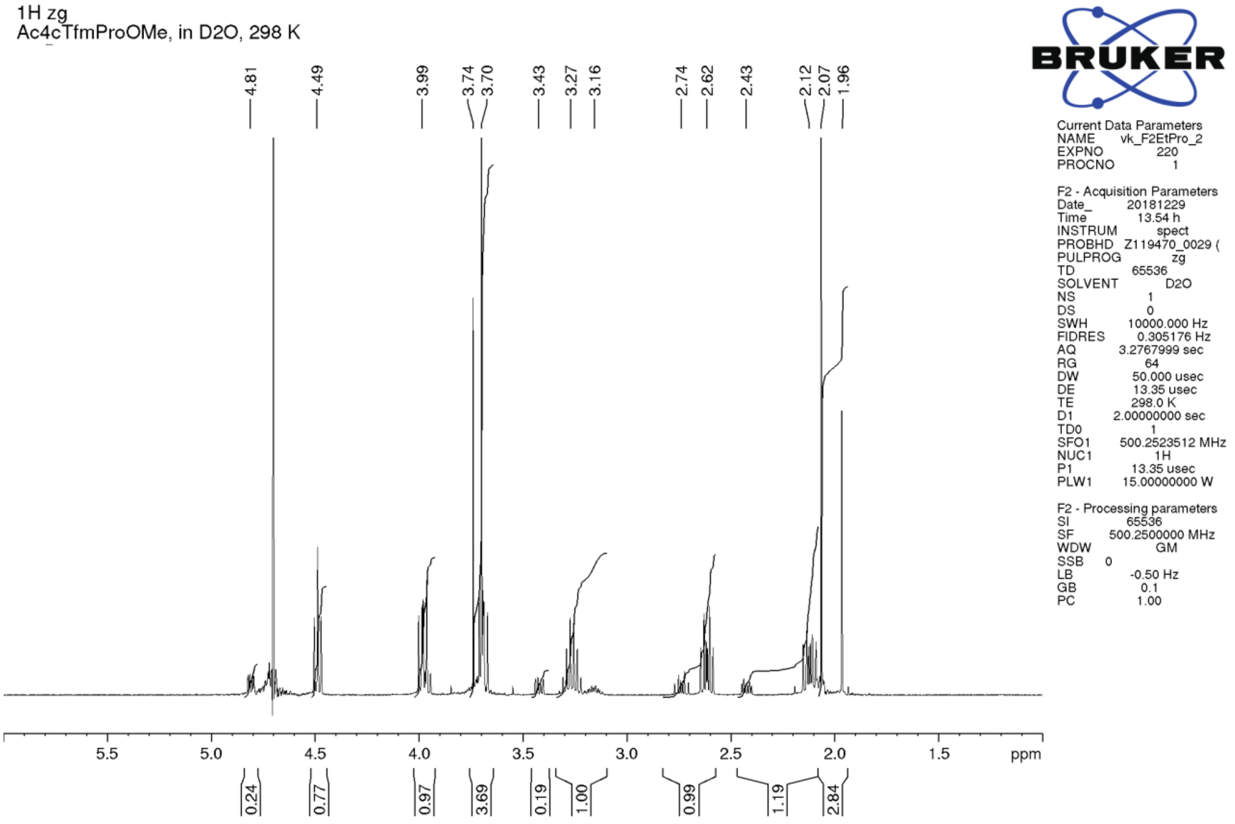

^{19}F NMR (471 MHz, D_2O):

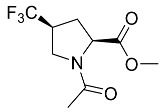

s-*trans*: −71.01 (d, *J* = 8 Hz);

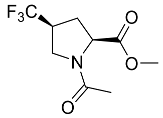

s-*cis*: −71.35 (d, *J* = 10 Hz).

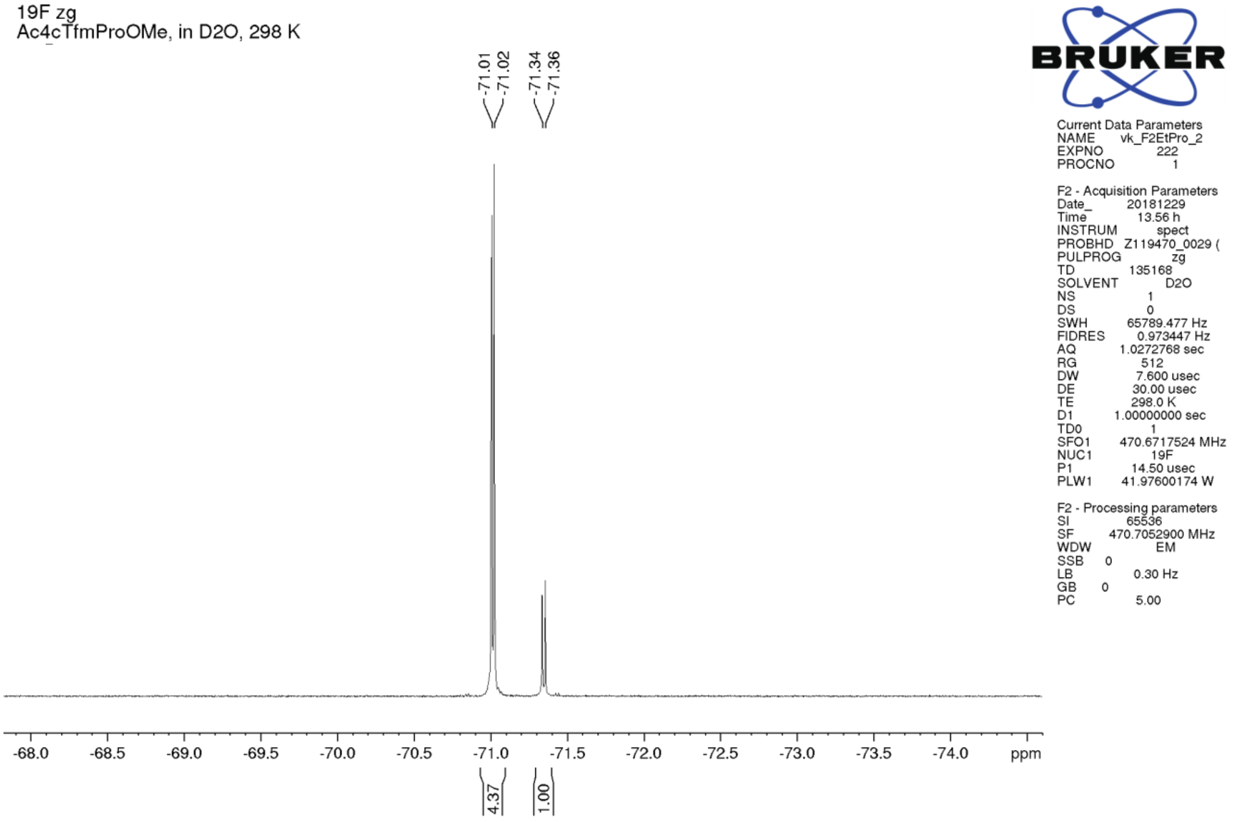

Methyl (*2R,4S*)-1-acetyl-4-trifluoromethylprolinate (**4**)

^{1}H NMR (500 MHz, D_2O), two rotamers, $K_{trans/cis}$ = 3.57 ± 0.13

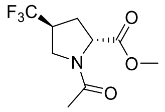

s-*trans*: 4.56 d, J = 9.2, 4.0 Hz, 1H, α-CH), 3.93 (dd, J = 11.1, 8.3 Hz, 1H, δ-CH), 3.70 (s, 3H, CH_3O), 3.69 (dd, J = 11.1, 9.0 Hz, 1H, δ-CH), 3.27 (m, 1H, γ-CH), 2.62 (dt, J = 13.4, 8.4 Hz, 1H, β-CH), 2.12 (dt, J = 13.4, 8.6 Hz, 1H, β-CH), 2.06 (s, 3H, Ac);

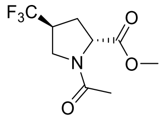

s-*cis*: 4.81 (dd, J = 8.6, 2.6 Hz, 1H, α-CH), 3.75 (s, 3H, CH_3O), 3.66 (m, *2*H, δ-CH_2), 3.19 (m, 1H, γ-CH), 2.50 (m, *2*H, β-CH_2), 1.97 (s, 3H, Ac).

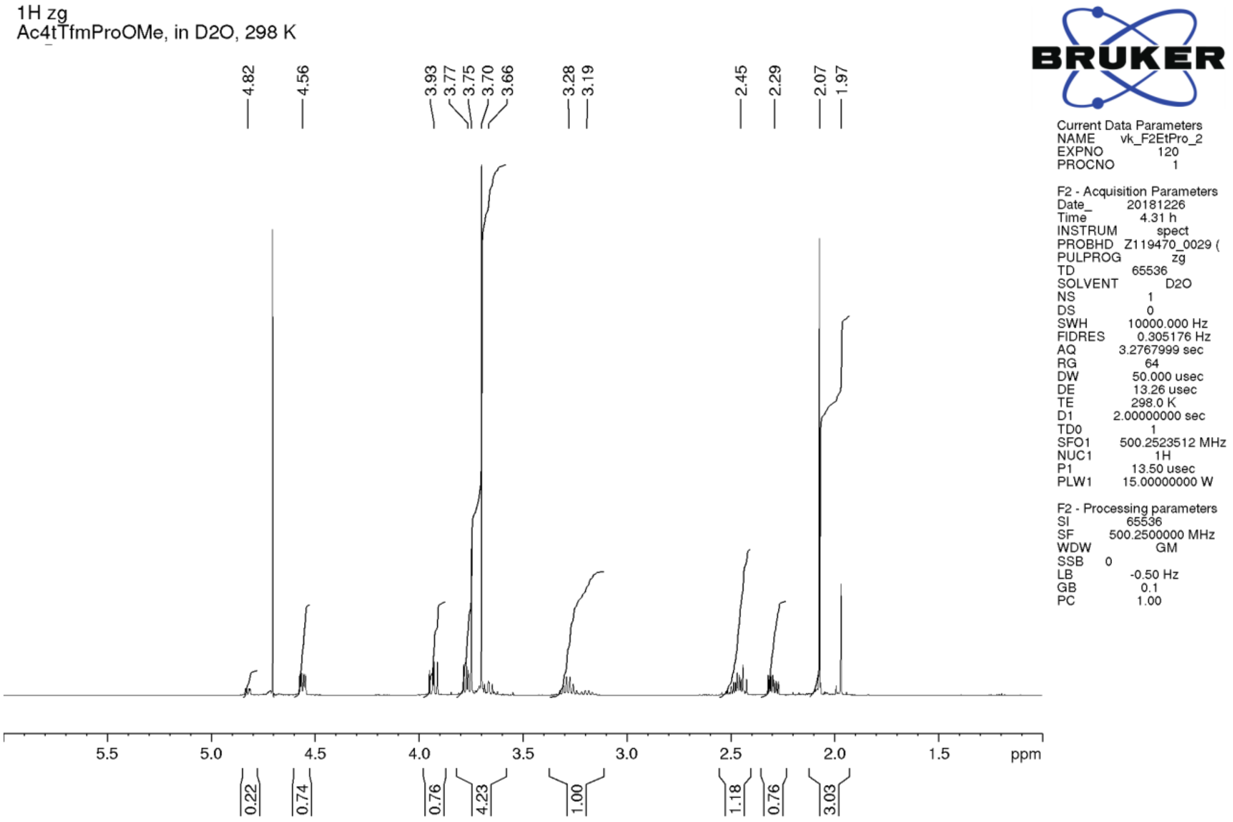

$^{13}C\{^1H\}$ NMR (126 MHz, D_2O):

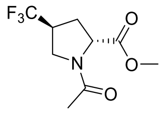

s-*trans*: 173.8 (s, CO_2Me), 173.1 (s, C(=O)N), 126.3 (q, J = 278 Hz, CF_3), 58.4 (s, α-CH), 53.1 (s, CH_3O), 47.0 (d, J = 4 Hz, δ-CH_2), 41.2 (q, J = 29 Hz, γ-CH), 28.2 (s, β-CH_2), 21.1 (s, Ac);

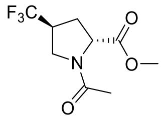

s-*cis*: 173.4 (s, C(=O)N), 173.3 (s, CO_2Me), 126.4 (q, J = 276 Hz, CF_3), 60.1 (s, α-CH), 53.4 (s, CH_3O), 45.5 (d, J = 4 Hz, δ-CH_2), 39.5 (q, J = 29 Hz, γ-CH), 29.6 (s, β-CH_2), 20.9 (s, Ac).

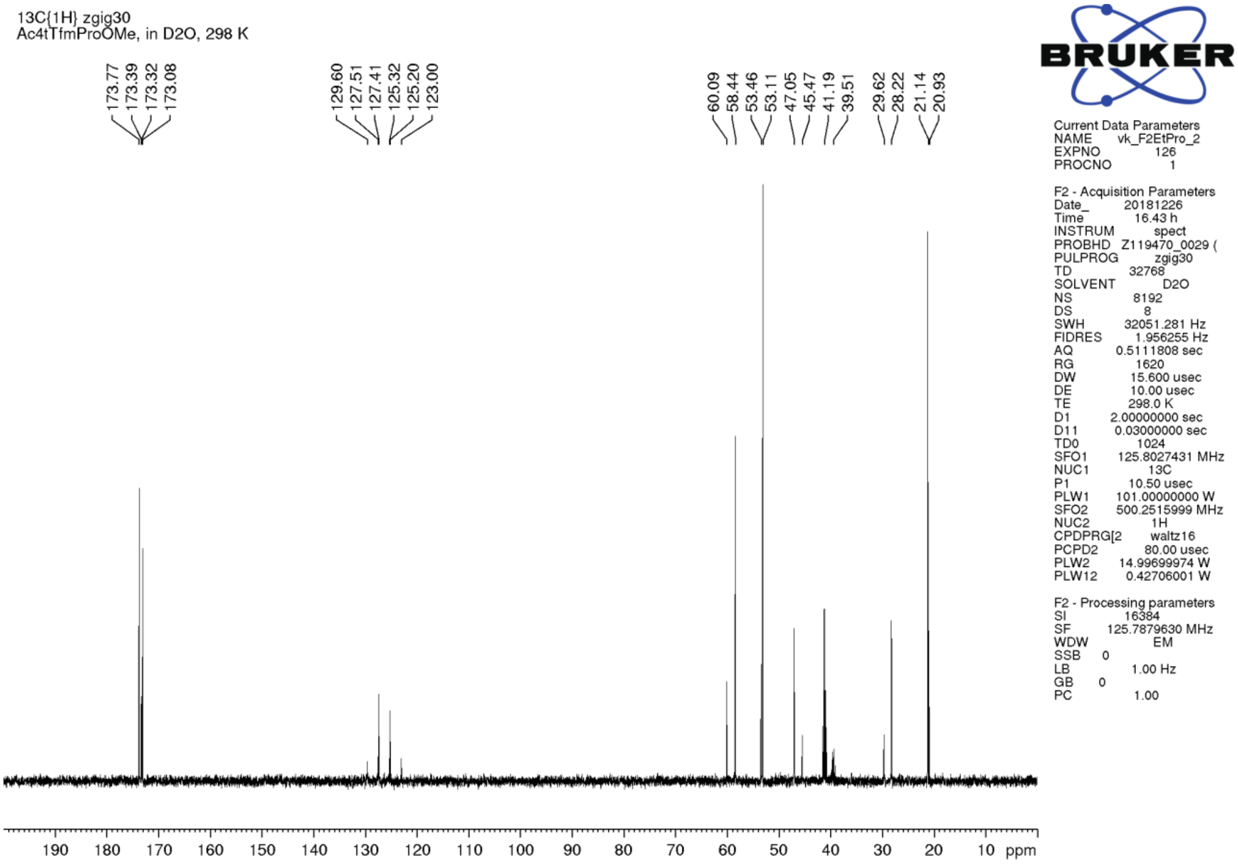

^{19}F NMR (471 MHz, D_2O):

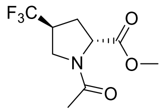

s-*trans*: −71.44 (d, *J* = 9 Hz);

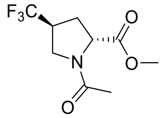

s-*cis*: −71.48 (d, *J* = 8 Hz).

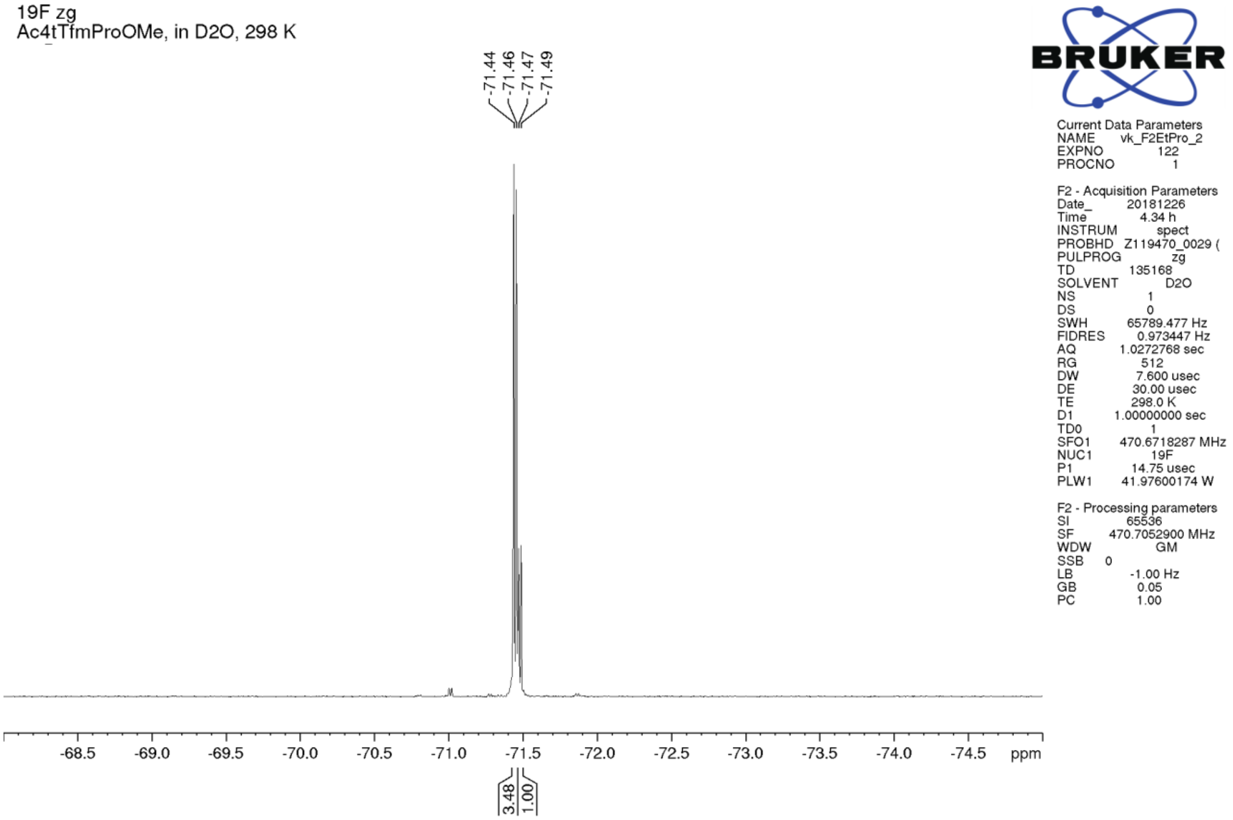

Methyl (*3r,6s*)-5-acetyl-1,1-difluoro-5-azaspiro[2.4]heptane-6-carboxylate (**5**)

^{1}H NMR (500 MHz, D_2O), two rotamers, $K_{trans/cis}$ = 3.94 ± 0.03

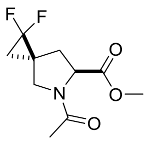

s-*trans*: 4.57 (dd, *J* = 8.8. 5.0 Hz, 1H, α-CH), 3.84 (dd, *J* = 11.1, 1.4 Hz, 1H, δ-CH), 3.71 (s, 3H, CH_3O), 3.68 (dd, *J* = 11.1, 4.8 Hz, 1H, δ-CH), 2.56 (ddd, *J* = 13.6, 9.0, 1.0 Hz, 1H, β-CH), 2.06 (dt, *J* = 13.9, 4.8 Hz, β-CH), 2.04 (s, 3H, Ac), 1.50 (m, 2H, CH_2);

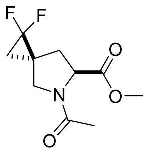

s-*cis*: 4.86 (dd, J= 8.7, 2.8 Hz, 1H, α-CH), 3.81 (dd, *J* = 12.3, 2.0 Hz, δ-CH), 3.76 (s, 3H, CH_3O), 3.37 (dd, *J* = 12.2, 4.3 Hz, 1H, δ-CH), 2.73 (ddd, *J* = 13.7, 8.8, 2.0 Hz, 1H, β-CH), 2.12 (dt, *J* = 13.6, 2.6 Hz, 1H, β-CH), 1.97 (s, 3H, Ac), 1.45 (m, 2H, CH_2).

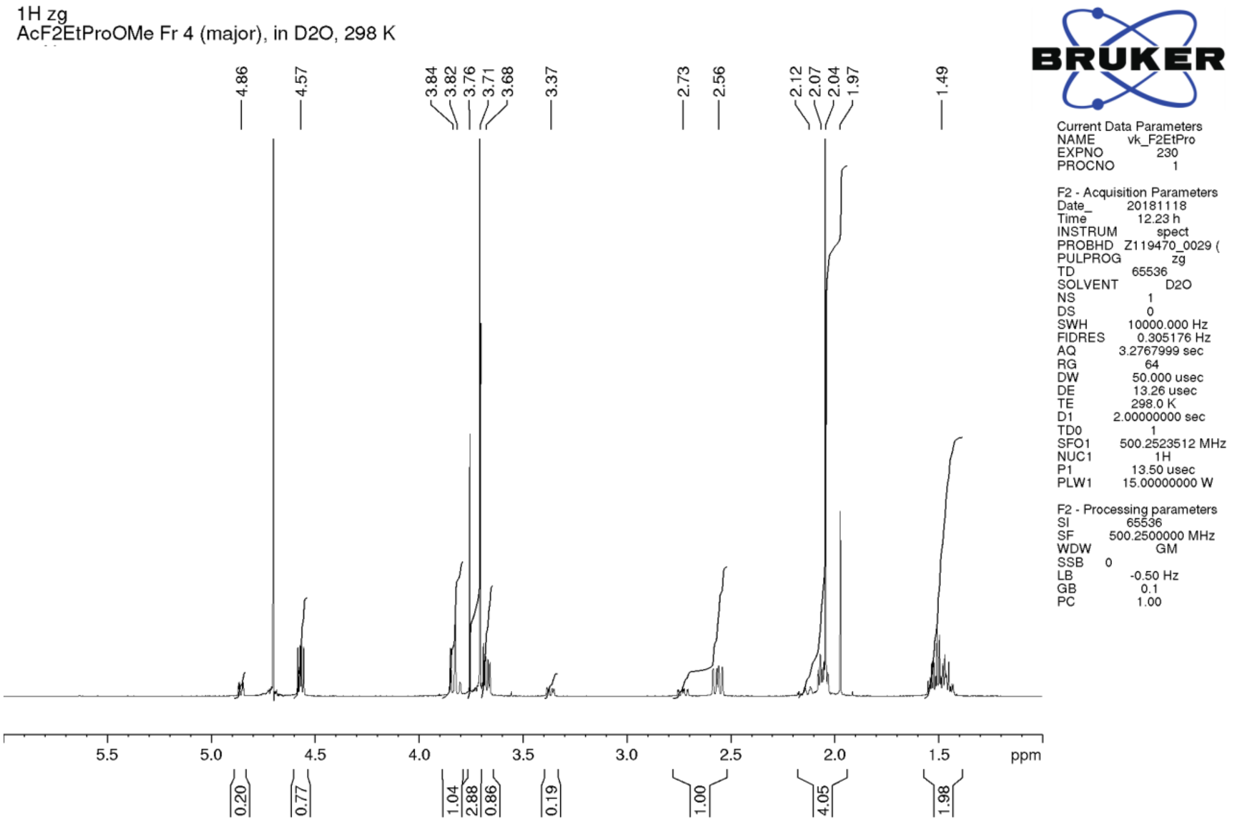

$^{13}C\{^{1}H\}$ NMR (126 MHz, D_2O):

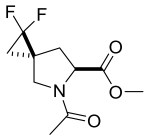

s-*trans*: 174.1 (s, CO_2Me), 173.0 (s, C(=O)N), 112.7 (t, CF_2, J = 287 Hz), 59.0 (s, α-CH), 53.1 (s, CH_3O), 50.3 (d, J = 6 Hz, δ-CH_2), 31.6 (d, J = 4 Hz, β-CH_2), 29.5 (t, J = 10 Hz, γ-C), 21.0 (s, Ac), 19.3 (t, J = 10 Hz, CH_2);

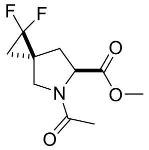

s-*cis*: 173.8 (s, CO_2Me), 173.3 (s, C(=O)N), 112.6 (t, CF_2, J = 287 Hz), 60.7 (s, α-CH), 53.4 (s, CH_3O), 49.4 (d, J = 4 Hz, δ-CH_2), 33.5 (s, β-CH_2), 28.2 (t, J = 12 Hz, γ-C), 20.8 (s, Ac), 20.8 (m, CH_2).

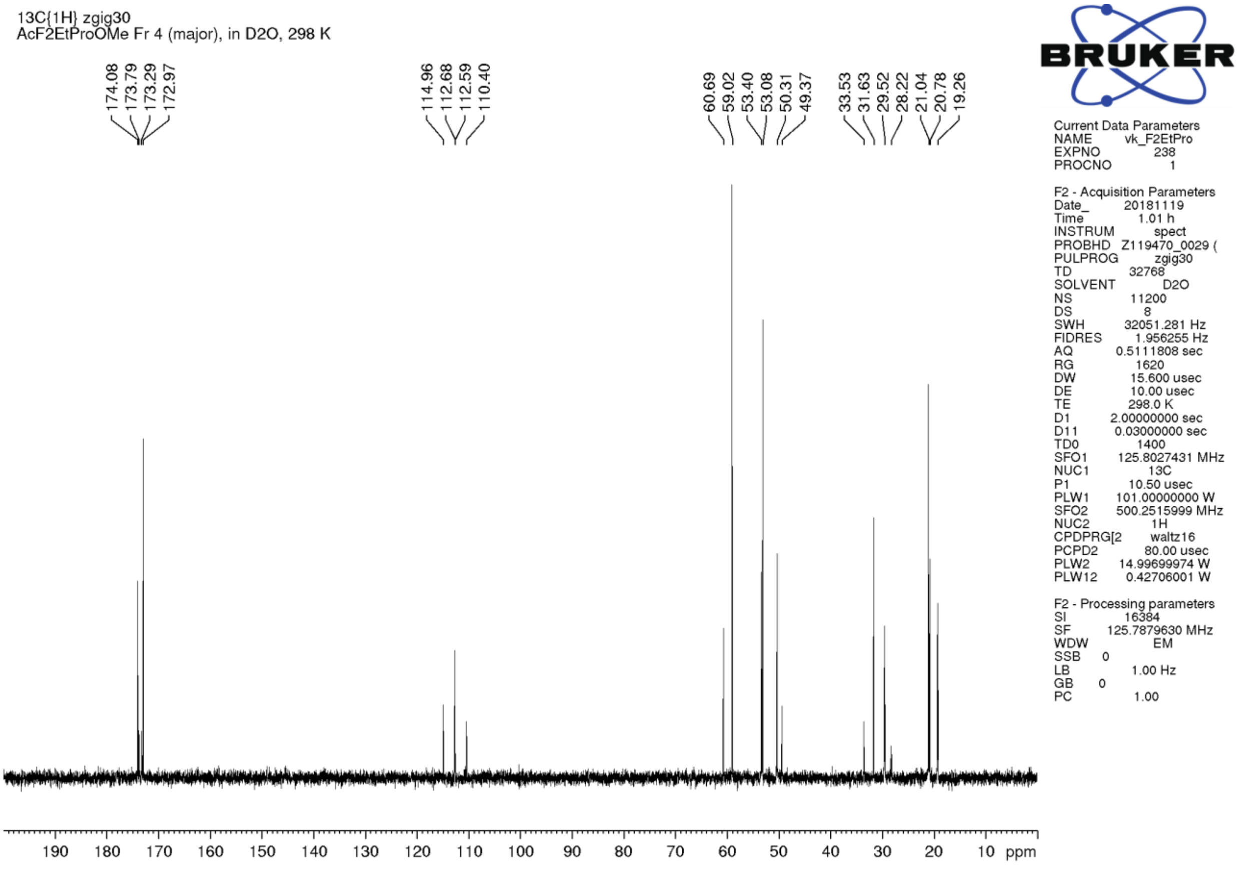

$^{19}F\{^{1}H\}$ NMR (471 MHz, D_2O):

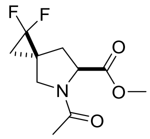

s-*trans*: −137.6 (d, *J* = 157 Hz), −138.5 (d, *J* = 157 Hz);

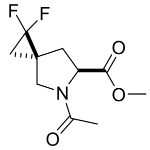

s-*cis*: −136.6 (d, *J* = 159 Hz), −137.3 (d, *J* = 159 Hz).

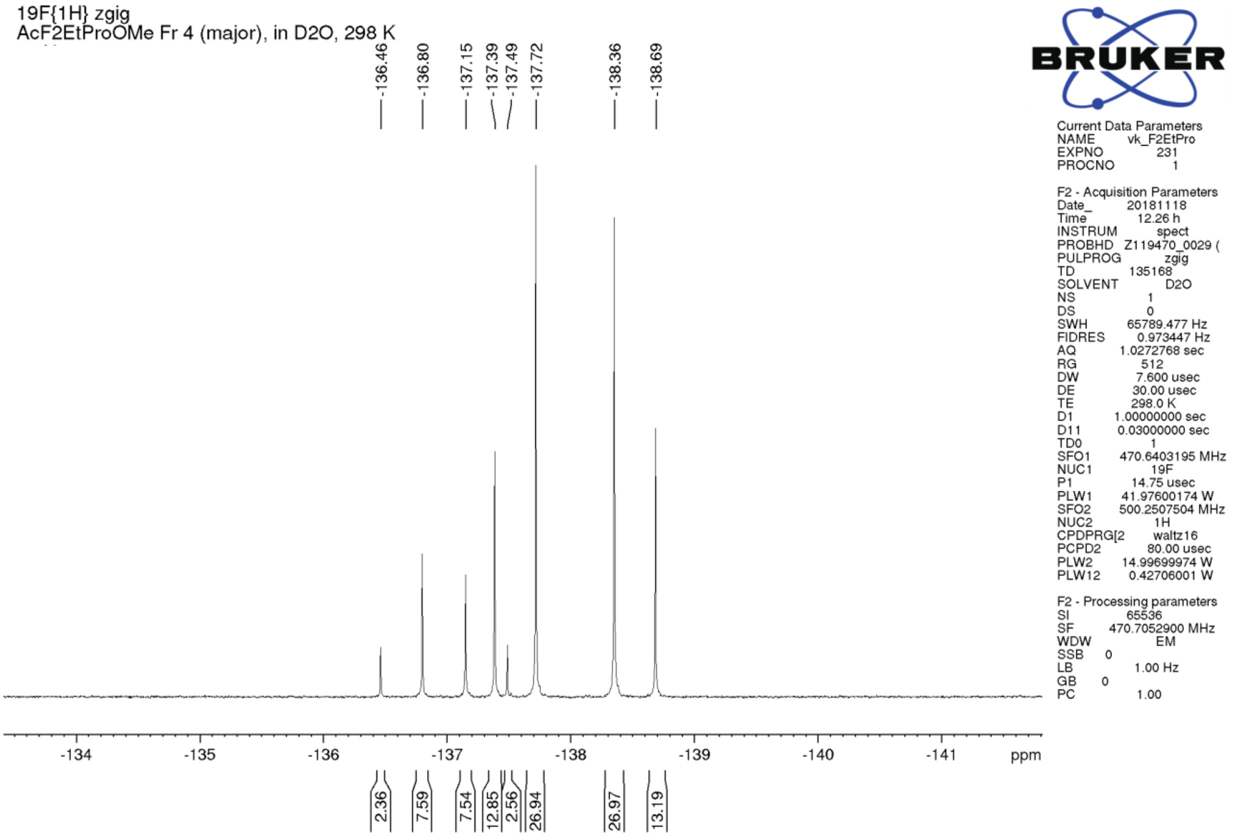

Methyl (*3r,6r*)-5-acetyl-1,1-difluoro-5-azaspiro[2.4]heptane-6-carboxylate (**6**)

^{1}H NMR (500 MHz, D_2O), two rotamers, $K_{trans/cis}$ = 3.15 ± 0.05

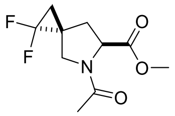

s-*trans*: 4.63 (dd, *J* = 9.1. 1.2 Hz, 1H, α-CH), 3.75 (ddd, *J* = 10.9, 3.8, 2.3 Hz, 1H, δ-CH), 3.70 (s, 3H, CH_3O), 3.69 (d, *J* = 10.5 Hz, 1H, δ-CH), 2.52 (ddm, *J* = 13.9, 9.2 Hz, 1H, β-CH), 2.06 (d, *J* = 14.6 Hz, 1H, β-CH), 2.05 (s, 3H, Ac), 1.54 (t, *J* = 9.0 Hz, 2H, CH_2);

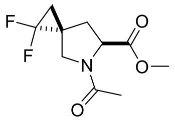

s-*cis*: 4.81 (d, *J* = 8.6 Hz, 1H, α-CH), 3.75 (s, 3H, CH_3O), 3.57 (d, *J* = 12.1 Hz, δ-CH), 3.53 (d, *J* = 12.2, 1H, δ-CH), 2.63 (ddd, *J* = 13.7, 8.8, 6.0 Hz, 1H, β-CH), 2.16 (d, *J* = 13.9 Hz, 1H, β-CH), 1.96 (s, 3H, Ac), 1.54 (m, 2H, CH_2).

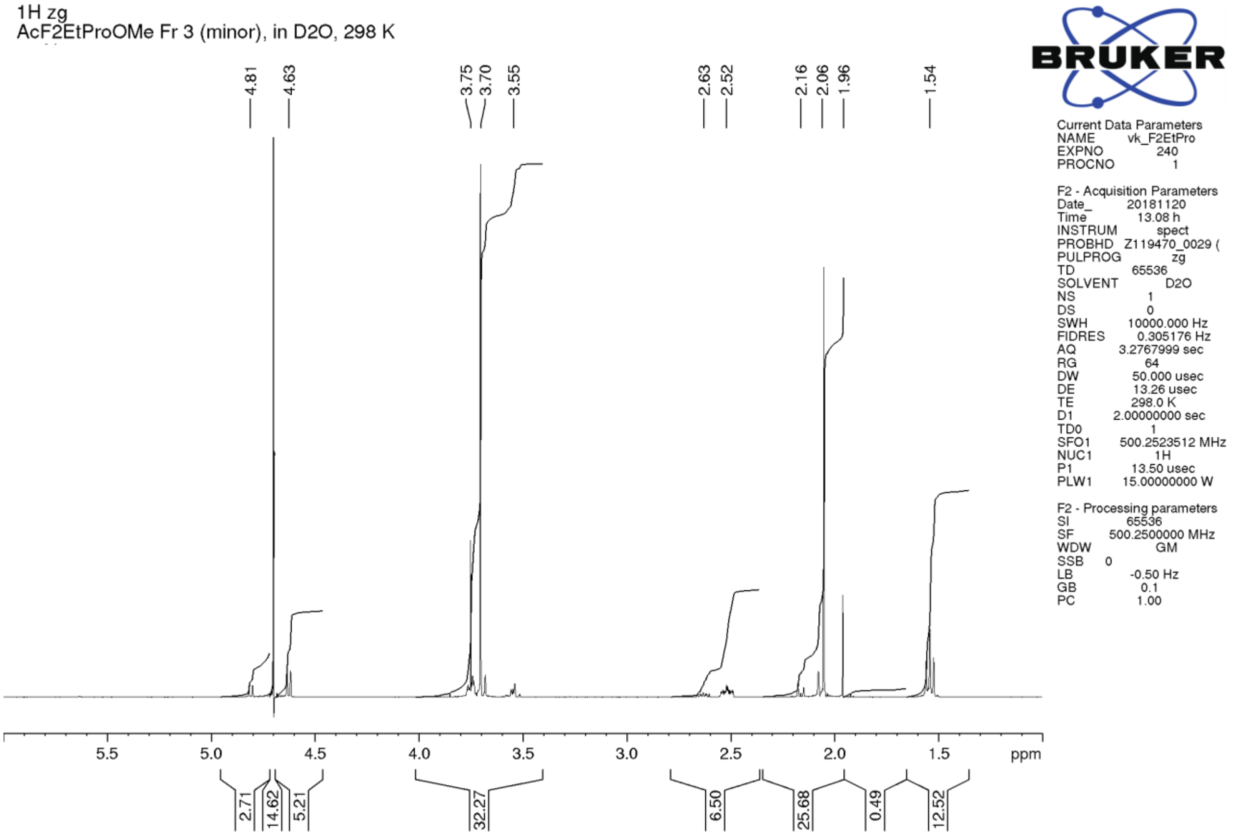

$^{13}C\{^1H\}$ NMR (126 MHz, D_2O):

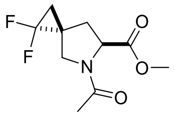

s-*trans*: 174.0 (s, CO_2Me), 173.1 (s, C(=O)N), 113.2 (t, CF_2, J = 288 Hz), 58.8 (s, α-CH), 53.1 (s, CH_3O), 48.8 (d, J = 4 Hz, δ-CH_2), 30.9 (s, β-CH_2), 29.0 (t, J = 10 Hz, γ-C), 21.0 (s, Ac), 15.5 (t, J = 11 Hz, CH_2);

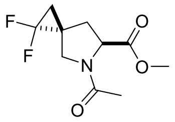

s-*cis*: 173.8 (s, CO_2Me), 173.2 (s, C(=O)N), 113.2 (t, CF_2, J = 287 Hz), 60.7 (s, α-CH), 53.5 (s, CH_3O), 47.2 (d, J = 6 Hz, δ-CH_2), 32.5 (d, J = 5 Hz, β-CH_2), 27.7 (t, J = 10 Hz, γ-C), 20.9 (s, Ac), 15.2 (t, J = 12 Hz, CH_2).

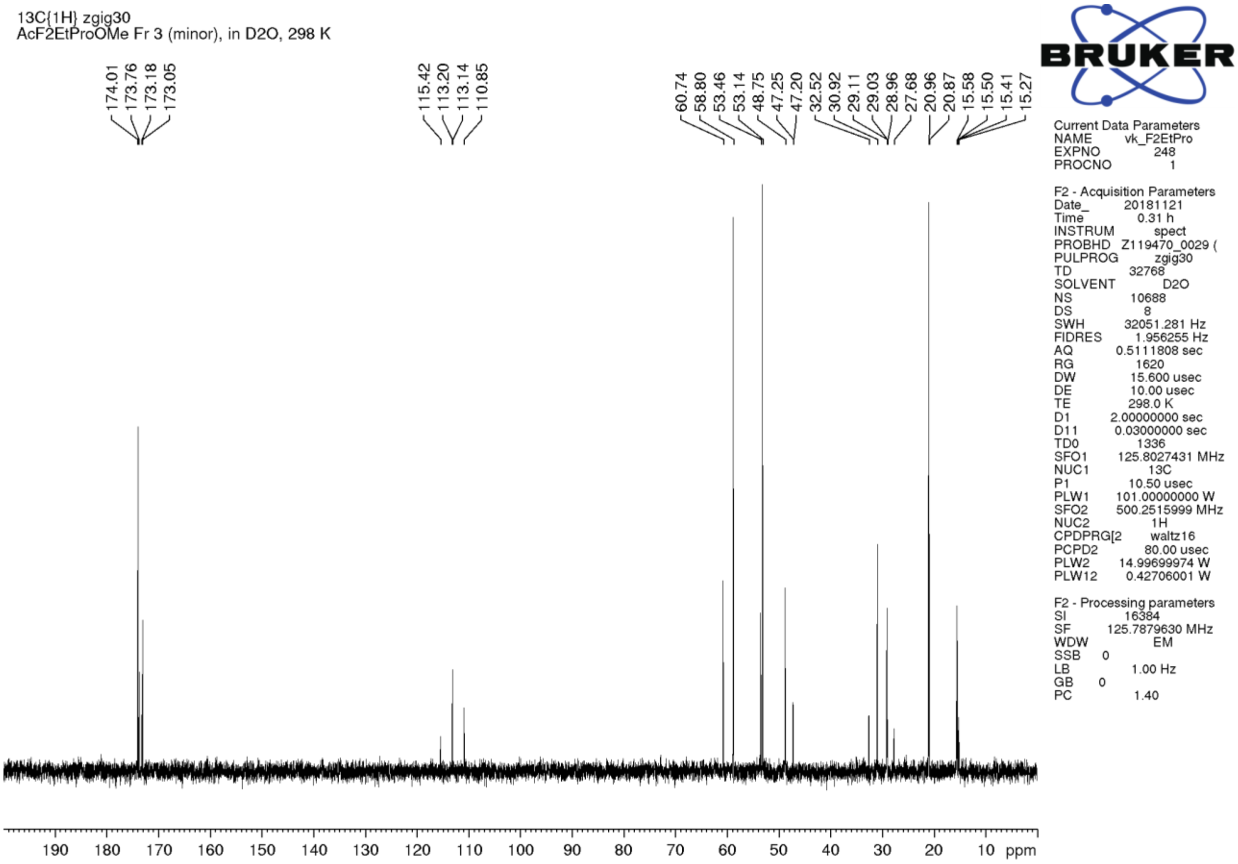

$^{19}F\{^1H\}$ NMR (471 MHz, D_2O):

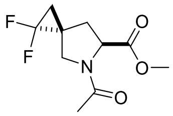

s-*trans*: −139.9 (d, *J* = 152 Hz), −140.1 (d, *J* = 150 Hz);

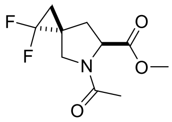

s-*cis*: −140.3 (d, *J* = 150 Hz), −140.5 (d, *J* = 151 Hz).

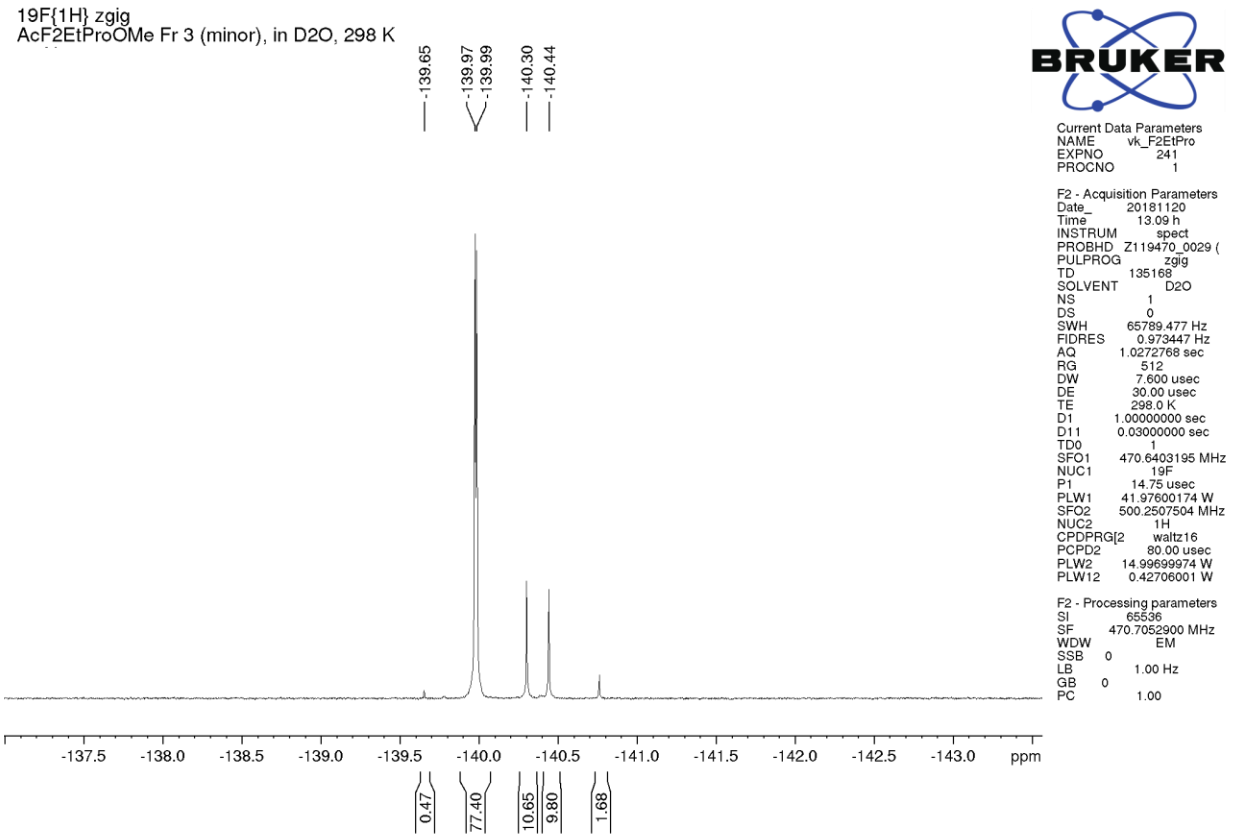

Methyl (6*S*)-5-acetyl-5-azaspiro[2.4]heptane-6-carboxylate (**7**)

^{1}H NMR (700 MHz, D_2O), two rotamers, $K_{trans/cis}$ = 3.92 ± 0.04

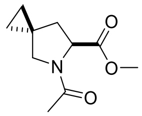

s-*trans*: 4.55 (dd, *J* = 8.8. 3.6 Hz, 1H, α-CH), 3.69 (s, 3H, CH_3O), 3.59 (d, *J* = 10.3 Hz, 1H, δ-CH), 3.38 (d, *J* = 10.3 Hz, 1H, δ-CH), 2.32 (dd, *J* = 13.0, 8.8 Hz, 1H, β-CH), 2.01 (s, 3H, Ac), 1.78 (dd, *J* = 13.1, 3.8 Hz, 1H, β-CH), 0.60-0.40 (m, 4H, $2xCH_2$);

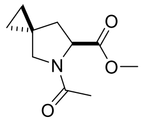

s-*cis*: 4.75 (d, *J* = 8.4 Hz, 1H, α-CH), 3.74 (s, 3H, CH_3O), 3.53 (d, *J* = 11.2 Hz, δ-CH), 3.11 (d, *J* = 11.5, 1H, δ-CH), 2.53 (dd, *J* = 12.9, 8.6 Hz, 1H, β-CH), 1.95 (s, 3H, Ac), 1.80 (d, *J* = 13.8 Hz, 1H, β-CH), 0.60-0.40 (m, 4H, $2xCH_2$).

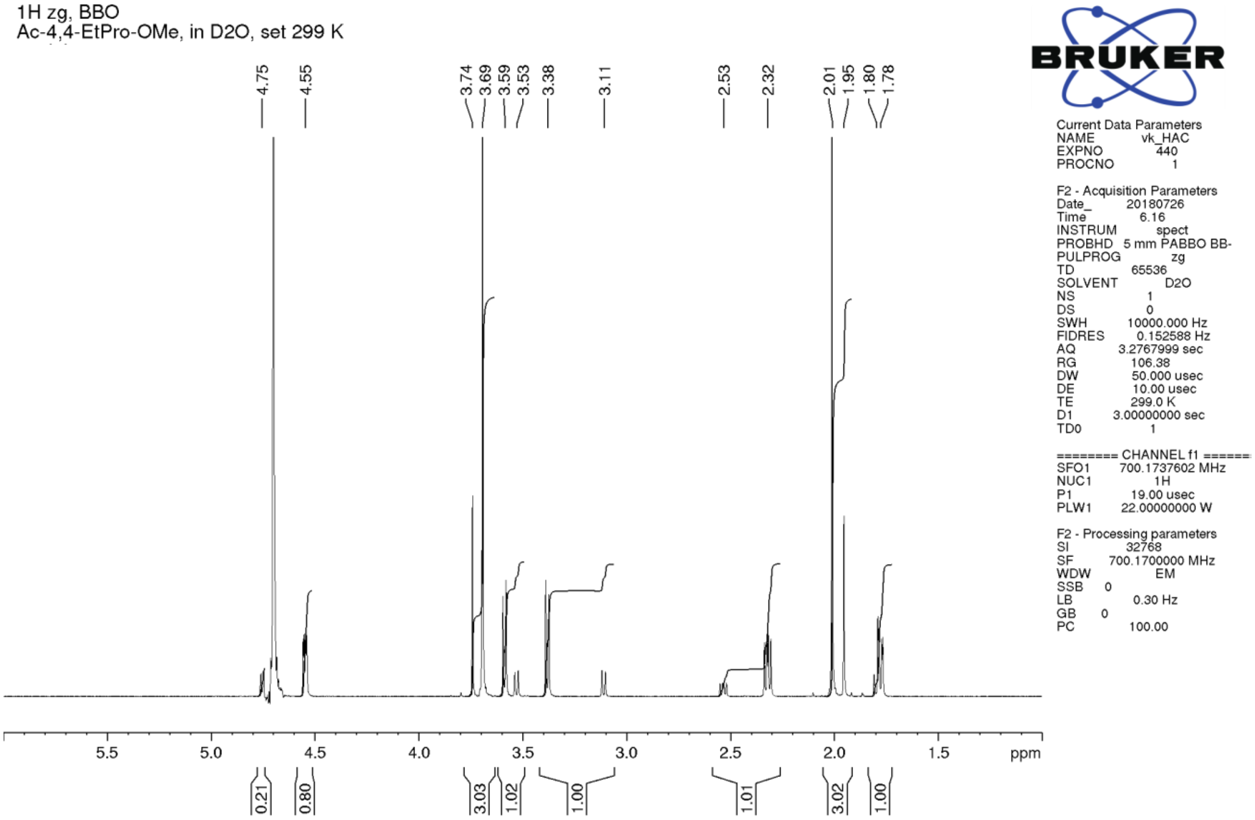

$^{13}C\{^1H\}$ NMR (176 MHz, D_2O):

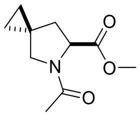

s-*trans*: 174.8 (s, CO_2Me), 173.0 (s, C(=O)N), 59.2 (s, α-CH), 55.3 (s, δ-CH_2), 52.9 (s, CH_3O), 36.9 (s, β-CH_2), 21.0 (s, Ac), 20.4 (s, γ-C), 11.6 (s, CH_2), 6.9 (s, CH_2);

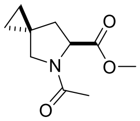

s-*cis*: 174.7 (s, CO_2Me), 173.3 (s, C(=O)N), 61.2 (s, α-CH), 53.6 (s, δ-CH_2), 53.2 (s, CH_3O), 38.3 (s, β-CH_2), 20.8 (s, Ac), 18.9 (s, γ-C), 13.3 (s, CH_2), 5.1 (s, CH_2).

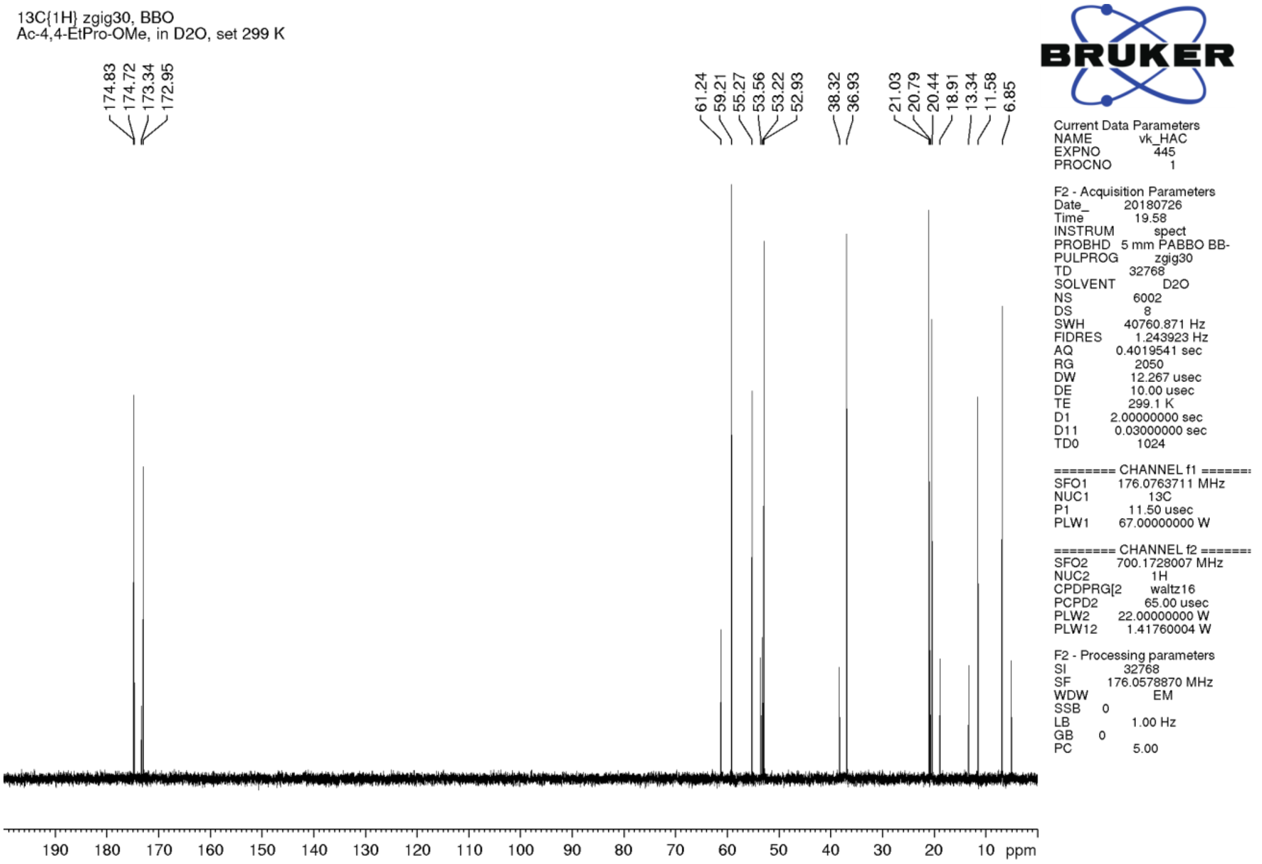

References

[S1] Kubyshkin, V.; Pridma, S.; Budisa, N. *New J. Chem.* **2018**, *42*, 13461-13470. DOI: 10.1039/C8NJ02631A
[S2] Kubyshkin, V.; Afonin, S.; Kara, S.; Budisa, N.; Mykhailiuk, P.K.; Ulrich, A.S. *Org. Biomol. Chem.* **2015**, *13*, 3171-3181. DOI: 10.1039/C5OB00034C
[S3] Bychek, R. M.; Levterov, V. V.; Sadkova, I. V.; Tolmachev, A. A.; Mykhailiuk, P. K. *Chem. Eur. J.* **2018**, *24*, 12291-12297. DOI: 10.1002/chem.201705708
[S4] Kubyshkin, V.; Durkin, P.; Budisa, N. *New J. Chem.* **2016**, *40*, 5209-5220. DOI: 10.1039/C5NJ03611A

[S5] Panasik, N., Jr.; Eberhardt, E. S.; Edison, A. S.; Powell, D. R.; Raines, R. T. *Int. J. Pept. Protein Res.***1994**, 44, 262−269. DOI: 10.1111/j.1399-3011.1994.tb00169.x
[S6] Renner, C.; Alefelder, S.; Bae, J. H.; Budisa, N.; Huber, R.; Moroder, L. *Angew. Chem. Int. Ed.* **2001**, 40, 923-925. DOI: 10.1002/1521-3773(20010302)40:5<923::AID-ANIE923>3.0.CO;2-%23
